# ER ribosomal-binding protein 1 regulates blood pressure and potassium homeostasis by modulating intracellular renin trafficking

**DOI:** 10.1186/s12929-023-00905-7

**Published:** 2023-02-19

**Authors:** Chu-Hsuan Chiu, Chin-Feng Hsuan, Shih-Hua Lin, Yi-Jen Hung, Chii-Min Hwu, Siow-Wey Hee, Shu-Wha Lin, Sitt-Wai Fong, Patrick Ching-Ho Hsieh, Wei-Shun Yang, Wei-Chou Lin, Hsiao-Lin Lee, Meng-Lun Hsieh, Wen-Yi Li, Jou-Wei Lin, Chih-Neng Hsu, Vin-Cent Wu, Gwo-Tsann Chuang, Yi-Cheng Chang, Lee-Ming Chuang

**Affiliations:** 1 Graduate Institute of Medical Genomics and Proteomics, National Taiwan University, Taipei, 100 Taiwan; 2 Institute of Biomedical Sciences, Academia Sinica, Taipei, 115 Taiwan; 3 Division of Cardiology, Department of Internal Medicine, E-Da Hospital, Kaohsiung, 824410 Taiwan; 4 Division of Cardiology, Department of Internal Medicine, E-Da Dachang Hospital, Kaohsiung, 82445 Taiwan; 5 School of Medicine, College of Medicine, I-Shou University, Kaohsiung, 840203 Taiwan; 6 Graduate Institute of Medical Science, National Defense Medical Center, Taipei, 114 Taiwan; 7 Section of General Medicine, Department of Medicine, Taipei Veterans General Hospital, Taipei, 111 Taiwan; 8 Division of Endocrinology and Metabolism, Department of Internal Medicine, Tri-Service General Hospital, National Defense Medical Center, Taipei, 100 Taiwan; 9 Faculty of Medicine, National Yang-Ming University School of Medicine, Taipei, 112 Taiwan; 10 Division of Nephrology, Department of Medicine, Tri-Service General Hospital, Taipei, 114 Taiwan; 11 Division of Endocrinology and Metabolism, Department of Internal Medicine, National Taiwan University Hospital, Taipei, 100 Taiwan; 12 Division of Genomic Medicine, Research Center for Medical Excellence, Transgenic Mouse Models Core, National Taiwan University, Taipei, 100 Taiwan; 13 Division of Nephrology, Department of Internal Medicine, National Taiwan University Hospital, Hsin-Chu Branch, Hsin-Chu, 302 Taiwan; 14 Department of Pathology, National Taiwan University Hospital, Taipei, 100 Taiwan; 15 Department of Medicinal Chemistry, College of Pharmacy, University of Florida, Gainesville, FL 32610 USA; 16 Division of Nephrology, Department of Internal Medicine, National Taiwan University Hospital Yunlin Branch, Yunlin, 640 Taiwan; 17 Division of Cardiology, Department of Internal Medicine, National Taiwan University Hospital Yunlin Branch, Yunlin, 640 Taiwan; 18 Division of Nephrology, Department of Internal Medicine, National Taiwan University Hospital, Taipei, 100 Taiwan; 19 Department of Pediatrics, National Taiwan University Hospital, College of Medicine, National Taiwan University, Taipei, 100 Taiwan; 20 Graduate Institute of Molecular Medicine, National Taiwan University, Taipei, 100 Taiwan; 21 Graduate Institute of Clinical Medicine, National Taiwan University, Taipei, 100 Taiwan

**Keywords:** Arrhythmia, Blood pressure, Cardiovascular disease, Hyperkalemia, Hyporeninemic hypoaldosteronism, Renin–angiotensin–aldosterone system, RRBP1

## Abstract

**Background:**

Genome-wide association studies (GWASs) have linked *RRBP1* (ribosomal-binding protein 1) genetic variants to atherosclerotic cardiovascular diseases and serum lipoprotein levels. However, how RRBP1 regulates blood pressure is unknown.

**Methods:**

To identify genetic variants associated with blood pressure, we performed a genome-wide linkage analysis with regional fine mapping in the Stanford Asia–Pacific Program for Hypertension and Insulin Resistance (SAPPHIRe) cohort. We further investigated the role of the *RRBP1* gene using a transgenic mouse model and a human cell model.

**Results:**

In the SAPPHIRe cohort, we discovered that genetic variants of the *RRBP1* gene were associated with blood pressure variation, which was confirmed by other GWASs for blood pressure. *Rrbp1*- knockout (KO) mice had lower blood pressure and were more likely to die suddenly from severe hyperkalemia caused by phenotypically hyporeninemic hypoaldosteronism than wild-type controls. The survival of *Rrbp1*-KO mice significantly decreased under high potassium intake due to lethal hyperkalemia-induced arrhythmia and persistent hypoaldosteronism, which could be rescued by fludrocortisone. An immunohistochemical study revealed renin accumulation in the juxtaglomerular cells of *Rrbp1*-KO mice. In the *RRBP1*-knockdown Calu-6 cells, a human renin-producing cell line, transmission electron and confocal microscopy revealed that renin was primarily retained in the endoplasmic reticulum and was unable to efficiently target the Golgi apparatus for secretion.

**Conclusions:**

RRBP1 deficiency in mice caused hyporeninemic hypoaldosteronism, resulting in lower blood pressure, severe hyperkalemia, and sudden cardiac death. In juxtaglomerular cells, deficiency of RRBP1 reduced renin intracellular trafficking from ER to Golgi apparatus. RRBP1 is a brand-new regulator of blood pressure and potassium homeostasis discovered in this study.

**Graphical Abstract:**

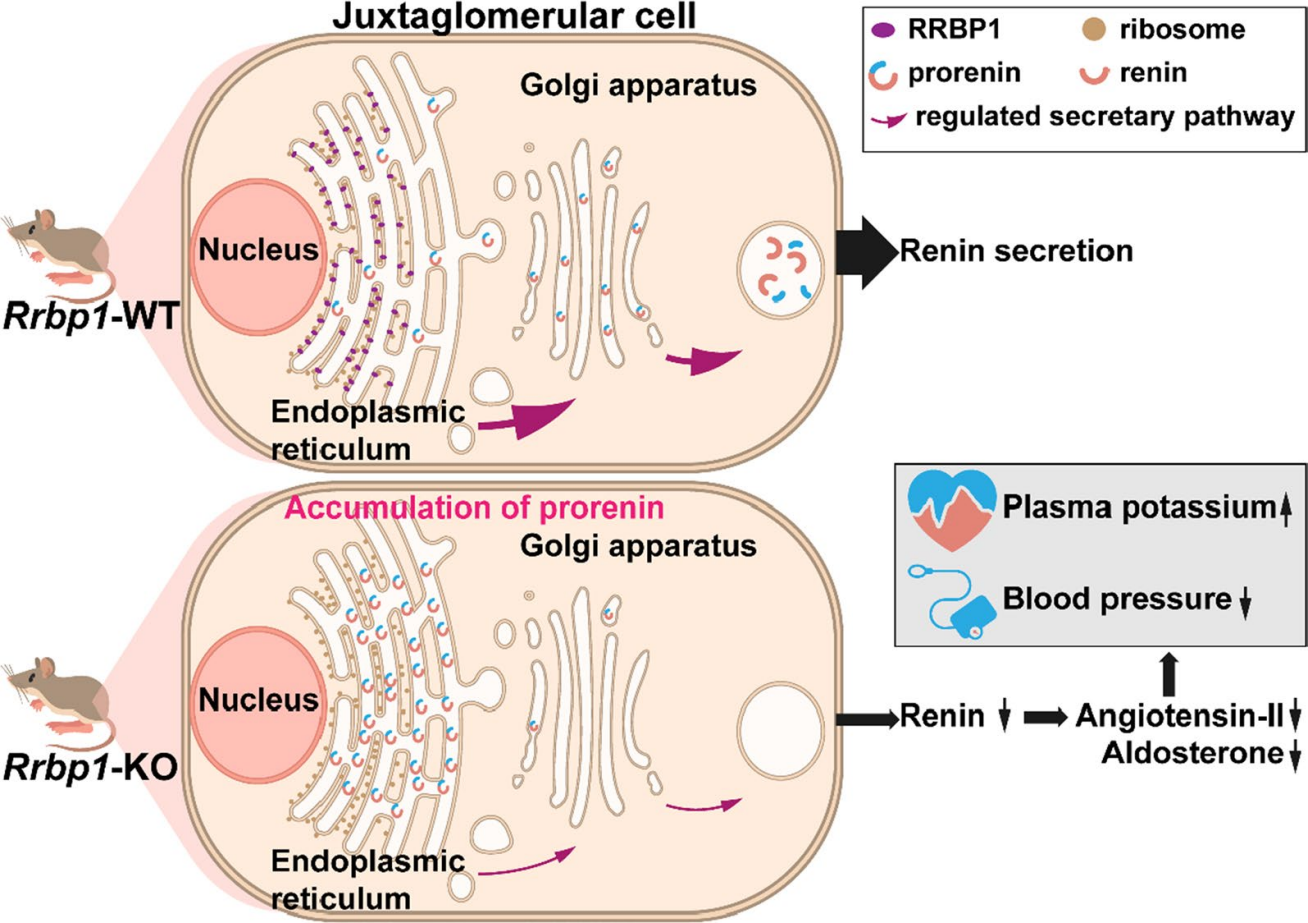

**Supplementary Information:**

The online version contains supplementary material available at 10.1186/s12929-023-00905-7.

## Background

Cardiovascular diseases (CVDs) are the leading cause of death worldwide; traditional risk factors include dyslipidemia, type 2 diabetes, tobacco use, and hypertension [1]. Several large genome-wide association studies (GWAS) have been carried out to identify genetic variants associated with CVDs and related diseases [2–4].

Recent GWASs have linked *RRBP1* (ER ribosomal-binding protein 1) genetic variants to intracranial arterial aneurysm, coronary artery disease, cardiovascular disease, and vascular headache [5–8]. However, how *RRBP1* regulates blood pressure is unknown. In this study, we discovered that *RRBP1* genetic variants were also associated with blood pressure in a large family-based genome-wide linkage and regional fine mapping of the Stanford Asia–Pacific Program for Hypertension and Insulin Resistance (SAPPHIRe) cohort. The associations were further validated in other GWASs for blood pressure. Consistently, we found that *Rrbp1*- knockout (KO) mice had lower blood pressure and hyporeninemic hypoaldosteronism, which cause severe hyperkalemic cardiac arrhythmia-induced sudden death.

Renin is a secretory protein that is synthesized and cleaved from prorenin in juxtaglomerular (JG) cells adjacent to the macula densa [9, 10]. Renin is the rate-limiting enzyme in the renin–angiotensin–aldosterone (RAAS) cascade [11]. Macula densa senses electrolytes concentration in the distal convoluted tubules and modulate renin secretion from JG cells in the kidney. Once renin is released into the bloodstream, renin converts angiotensinogen to angiotensin-I (Ang-I) in the liver [12]. Ang-I is further cleaved to angiotensin-II (Ang-II) by the angiotensin converting enzyme (ACE) in the lung [13]. Ang-II causes vasoconstriction and stimulates the adrenal gland to release aldosterone [14], which regulates blood volume by increasing sodium reabsorption, potassium excretion, and water reabsorption [14–16]. Moreover, the renin–angiotensin–aldosterone system is triggered by the release of the protease renin from the kidneys, which is then controlled by negative feedback loops.

RRBP1 was initially identified as an ER transmembrane protein that interacts with ribosomes [17–19], but it also mediates interactions between the endoplasmic reticulum (ER) and microtubules [20, 21] that transport nascent protein cargo from the ER to the Golgi apparatus. Furthermore, *RRBP1* deficiency can decrease the secretion of apolipoproteins E and collagen [20, 22, 23]. These findings suggest that RRBP1 is required for nascent protein translocation, intracellular trafficking, and secretion.

According to our preliminary findings, genetic variants of *RRBP1* were associated with blood pressure in human genetic studies. Next, we found that *Rrbp1*-KO mice display hypotension and hyporeninemic hypoaldosteronism. In this study, the aim was to investigate the molecular mechanisms by which RRBP1 regulates blood pressure and the RAAS axis.

## Methods

### Genome-wide linkage study

The Stanford Asia–Pacific Program for Hypertension and Insulin Resistance (SAPPHIRe) is a collaborative family study sponsored by the Family Blood Pressure Program of the National Heart, Lung and Blood Institute of the National Institutes of Health. The study was conducted to identify the genetic determinant of hypertension and insulin resistance in participants of Chinese ancestry. The study collected sibling pairs of over 1144 participants from 360 nuclear families who were either concordant or discordant for high blood pressure as previously described [24]. The definition of high blood pressure was systolic blood pressure > 140 mm Hg and diastolic blood pressure > 90 mm Hg or with two medications for hypertension. Blood pressure in the bottom 30% of age- and sex-adjusted blood pressure distributions was defined as low-normal blood pressure. Individuals with heart, liver, or kidney diseases or chronic diseases such as diabetes or cancer were excluded. The study was approved by the Institutional Review Boards/Research Ethics Review Committee including National Taiwan University Hospital, National Health Research Institutes, Taichung Veterans General Hospital, Taipei Veterans General Hospital, and Tri-Service General Hospital. All participants signed informed consent. All procedures were conducted according to principles outlined in the Declaration of Helsinki. The detailed analysis is shown in Additional file 1.

### Animals

To establish *Rrbp1*-KO mice, two targeting vectors were generated to delete exons 4–24 of the *Rrbp1* gene through recombineering [24, 25]; the detailed method and schematic of the knockout construct (Fig. S1A) are shown in Additional file 1. C57BL6/129 J mice of mixed genetic background mice were generated by breeding B6 with 129 J for up to six generations because conventional knockout mice of C57BL/6 background were difficult to acquire. Cross-breeding heterozygous with heterozygous of both C57BL6/129J mixed genetic background generated *Rrbp1*-WT (wild-type), *Rrbp1*-HE (heterozygotes), and *Rrbp1*-KO (homozygous knockout) mice for subsequent experiments. During the high-potassium loading mouse survival test, mice underwent high K^+^ intake and were intraperitoneally injected with fludrocortisone acetate once every 2 days for a total of 30 days. Death events were recorded. Mice were sacrificed for tissue dissection or euthanized by excess carbon dioxide administration. All animal protocols and experimental procedures were approved by the Institutional Animal Care and Utilization Committee, Academia Sinica (IACUC number: 19–07-1331) and performed according to the National Institutes of Health (NIH) guidelines for the Care and Use of Laboratory Animals.

### Telemetry ECG and blood pressure measurements

For non-invasive ECG recording, the 12–16-week-old mice were anesthetized using 2.5% isoflurane in pure oxygen at a flow rate of 0.5 L/min. Mice ECG were recorded and analyzed using Powerlab8/30 and Animal Bio Amp. For telemetry ECG recording, the 12–16-week-old mice were anesthetized using 2.5% isoflurane in pure oxygen at a flow rate of 0.5 L/min. The telemetric transmitters (ETA-F10, Data Sciences International) were implanted in the neck with electrodes that were tunneled subcutaneously as previously described [26]. Two-hour ECGs were recorded in conscious mice before and during high K^+^ intake for two days. Recordings were analyzed using Dataquest A.R.T. Software (Data Sciences International). Mice (10–16 weeks old, body weight 25–30 g) were placed in a plastic tube restrainer and rested for more than 10 min; this step was conducted every day for a week before recording for adaptive training. Each mouse was measured over 60 times. From 13:00–15:00, SBP, DBP and MBP were measured using a noninvasive tail-cuff blood pressure monitor (MOORLAB NIBP, MOOR).

### Blood and urine analysis

Ten to sixteen-week-old male and female mice were used. For high potassium loading test or the fludrocortisone acetate treatment assay, mice were placed in metabolic cages at 11:00 am. They were first fed a control diet (D10012Mi, Research Diets) and normal water for 24 h, followed by a high K^+^ diet (5% potassium was added as potassium chloride) and water containing 5% KCl for another 48 h. For fludrocortisone acetate treatment assay, mice were intraperitoneally injected with saline or 100 mg/kg fludrocortisone acetate (F6127, Sigma) after high K^+^ intake for 24 h. After another 24 h, urine and blood from the submandibular vein were collected and examined. Electrolyte profiles were analyzed with various cobas c111 analyzers (Roche). The plasma samples with a hemolysis index from zero to “+  + ” (indicated very mild level of hemolysis) were obtained to measure electrolytes and perform an ELISA assay. Tables S1–S3 show all raw data of electrolyte levels in blood and urine samples.

### Immunohistochemistry

Mouse kidneys and adrenal glands were paraffin-embedded and sectioned to 4 µm. Adrenal gland sections underwent standard hematoxylin and eosin (H&E) staining. Kidney sections were rehydrated by immersing in xylene followed by 100%, 95%, 70%, and 50% alcohol. Sections were immersed in 1 mM citric acid buffer (pH 6.0) with Tween-20 and heated to 92–95 °C for antigen retrieval. To quench endogenous peroxidase activity, sections were incubated within 3% H_2_O_2_ for 20 min before blocking with 3% BSA/PBST. Immunostaining was conducted using primary antibody for renin (1:20; H0005972-M01, Abnova), secondary antibodies HRP-anti-rabbit/mouse (K5007, Dako), and DAB chromogen solution. Stained sections were imaged with an advanced microscope (Axio Imager. A1, Zeiss). Additional file 1 details the phenotypic analysis methods.

### Calu-6 cell culture

The human renin-secreting Calu-6 cell line was purchased from ATCC (HTB-56™). Calu-6 cells were cultured in Dulbecco’s Modified Eagle Medium/Nutrient Mixture F-12 supplemented with 10% fetal bovine serum (FBS) and 1% penicillin–streptomycin (p4333, Sigma) and maintained at 37 °C in a 5% CO_2_-containing humidified incubator. Cells were cultured in 10-cm plastic tissue culture dishes (430,167, Corning Inc.) and harvested with trypsin upon reaching the logarithmic growth phase.

### Lentiviral transfection

Lentiviral knockdown *RRBP1* and lentiviral scramble control particles were purchased from RNAi (Academia Sinica, Taiwan). Calu-6 cells were infected at 45–55% confluency with lentiviral particles and 24 μg polybrene in 3 ml growth medium at a multiplicity of infection of 10. After 24 h, the medium was replaced with 4 ml fresh growth medium. After 48 h, the medium was replaced with 4 ml fresh growth medium and 8 μg puromycin (A1113802, Thermo Fisher) for cell selection. After 5 days, all transfected Calu-6 cells were passaged for other experiments. *RRBP1*-knockdown efficiency was assessed using real-time PCR (qPCR) and western blot.

### Western blot assay

Mouse tissues and cell lysates were extracted using RIPA lysis buffer (20–188, Millipore) according to the manufacturer’s protocol. Cell culture supernatant was collected and concentrated with Amicon Ultra-15 tubes (10 KDa, Millipore). The following antibodies were used: anti-RRBP1 (PA5-21,392, Invitrogen), anti-RRBP1 (Ab95983, Abcam), anti-RRBP1 (HPA011924, Sigma), anti-Hsp70 (DF2698, Affinity), anti-β-actin (GTX109697, Genetex), anti-renin (H0005972-M01, Abnova), anti-ACE (MA5-32,741, Invitrogen), anti-β-tubulin (tcaba2, Taiclone), anti-SGK1 (ab32374, Abcam), anti-ADCY5/6 (PA5-75,274, Abnova), and anti-calnexin (ab22595, Abcam). The blots were detected with Trident pico Western HRP Substrate (GTX17435, Genetex), and images were analyzed using a UVP BioSpectrum Imaging System.

### Real-time PCR (qPCR)

RNA samples were extracted with TRIzol® reagent (Life Technologies). Total RNA (2 µg) was reverse transcribed to cDNA using reverse transcriptase (HD Life Sciences). The qPCR assay was conducted with SYBR-Green qPCR mix (HD Life Sciences) and a LightCycler® 480 Real-Time PCR System (Roche).

### Immuno-electron microscopy

Cells were plated on plastic coverslips in 60-mm tissue culture plates. Samples were blocked and permeabilized with PBS containing 5% BSA and 0.1% saponin for 30 min followed by incubation with primary antibodies for renin (1:20; H0005972-M01, Abnova) in PBS containing 5% BSA and 0.05% saponin for two hours at room temperature. After four washes with PBS, samples were incubated with secondary antibody (1:100; 2002 Nanogold®-Fab, Nanoprobes) in PBS containing 5% BSA and 0.05% saponin for one hour at room temperature followed by fixation with 2% glutaraldehyde in PBS for 30 min at room temperature. Sections were examined using a Philips CM 100 transmission electron microscope at 80 kV and imaged with a Gatan Orius CCD camera.

### Immunofluorescence staining.

Cells were cultured on coverslips and then fixed in 10% formaldehyde solution (HT501128, Sigma) for 10 min at room temperature, followed by washing thrice with PBS for five minutes each. The cells were incubated overnight at 4 °C with the following primary antibodies in blocking buffer: mouse anti-renin (1:50, H00005972-M01, Abnova), rabbit anti-calnexin (1:400, ab22595, Abcam), rabbit anti-GOLIM4 (1:200, PAB28477, Abnova), and rabbit anti-RRBP1 (1:200, ab95983, Abcam) followed by Alexa Fluor® 488 goat anti-mouse IgG_(H+L)_ (1:100, A11029, Thermo Fisher) or Alexa Fluor™ 555 goat anti-rabbit IgG_(H+L)_ (1:100, A21428, Thermo Fisher) secondary antibody for 1.5 h at room temperature. Finally, slides were mounted with a drop of DAPI Fluoromount-G® (0100–20, Southern Biotech). Samples were examined using a laser scanning confocal microscope (Zeiss LSM 700) and imaged with a 63 × oil objective lens in a 1024 × 1024 pixel format at a 12-bit intensity resolution. Additional file 1 details the in vitro analysis.

### Statistical methods

All data are represented as mean ± standard error of the mean (SEM). Data that compared three independent groups (blood pressure of *Rrbp1*-WT, HE, and KO mice) underwent ordinary one-way ANOVA. The Kaplan–Meier survival curve and log-rank test were used for survival analysis. Datasets that followed a normal distribution were compared using an unpaired, two-tailed Student’s t-test (*p* ≤ 0.05). Datasets that did not follow a normal distribution were compared using a two-tailed Mann–Whitney U test (*p* ≤ 0.05). All representative images were selected from an experiment whose data best matched the average for each assay.

## Results

### *RRBP1* genetic variants are associated with blood pressure in the SAPPHIRe study

To identify genetic loci that influenced blood pressure, we performed a genome-wide linkage scan of 1144 participants from 360 nuclear families in the SAPPHIRe cohort and quantitatively mapped a trait locus located on chromosome 20 between 14.7–18.3 Mb. This region was further refined by performing a sliding window analysis to identify haplotypes associated with blood pressure. A 7-SNP haplotype numbered H2:2,211,121 (haplotype frequency: 16.1%) of the *RRBP1* gene containing the SNP rs6080761 was associated with lower systolic blood pressure (*Z* =  − 3.90, haplotype-specific* P* = 9.6 × 10^−5^, global *P* = 5.29 × 10^−3^), lower diastolic blood pressure (*Z* =  − 3.796, haplotype-specific* P* = 1.47 × 10^−5^, global *P* = 0.02), and lower mean blood pressure (*Z* =  − 4.135, haplotype-specific* P* = 3.6 × 10^−5^, global *P* = 5.12 × 10^−3^) (Table 1). In the International Consortium for Blood Pressure study (ICBP pha003588) that included 132,671 participants and found that rs6080761 was associated with diastolic blood pressure (*P* = 0.01) and mean blood pressure (*P* = 0.02). Similarly, in the National Heart, Lung, and Blood Institute (NHLBI, pha003048) Family Heart Study, rs6080761 was associated with diastolic blood pressure (*P* = 0.01) and mean blood pressure (*P* = 0.03) among 2,756 participants. This SNP was also associated with systolic blood pressure (*P* = 0.03) among 1,538 Caucasians in the Genetic Epidemiology Network of Arteriopathy study (GENOA pha00309.1).

**Table 1:** FBAT association analysis of RRBP1 7-SNP (rs7272683, rs2236255, rs6034875, rs6080761, rs6080765, rs8120179, rs3790308) haplotypes with blood pressure

Haplotype	Freq	Number of informative families	SBP		DBP		MBP	
			Z	*P* ^a^	Z	*P* ^a^	Z	*P* ^a^
H1: 2,211,111	0.211	91	1.043	0.297	0.755	0.450	0.963	0.335
H2: 2,211,121	0.161	78	− 3.901	9.6 × 10^−5^	− 3.796	1.47 × 10^−4^	− 4.135	3.6 × 10^−5^
H3: 2,111,111	0.131	62	1.652	0.099	1.470	0.142	1.648	0.099
H4: 1,222,211	0.079	45	1.260	0.208	1.853	0.064	1.793	0.073
H5: 2,111,121	0.071	46	− 1.956	0.051	− 0.785	0.433	− 1.464	0.143
H6: 1,122,221	0.054	34	0.772	0.440	0.545	0.585	0.653	0.514
H7: 1,222,221	0.050	39	1.020	0.308	0.669	0.504	0.909	0.364
H8: 1,122,211	0.043	35	1.223	0.221	0.432	0.666	0.841	0.400
H9: 1,211,122	0.025	15	1.470	0.141	1.569	0.117	1.669	0.095
Global *P*-value ^b^			5.29 × 10^−3^		0.02		5.12 × 10^−3^	

FBAT, family-based association test; SNP, single nucleotide polymorphism; Freq., frequency; SBP, systolic blood pressure; DBP, diastolic blood pressure; MBP, mean blood pressure

### *Rrbp1* knockout mice exhibit lower blood pressure and are prone to sudden death

We created *Rrbp1*-KO mice by knocking out the regions from exons 4 to exons 24 of the *Rrbp1* gene to determine how RRBP1 regulates blood pressure (Additional file 1: Fig. S1A and B). Immunoblots revealed that RRBP1 is primarily expressed in the intestine, liver, kidney, and pancreas of mice (Additional file 1: Fig. S1C). The *Rrbp1*-WT and *Rrbp1*-KO mice showed no significant differences in morphology, anatomy, body composition, body weight, or abdominal organ weight (Additional file 1: Fig. S2).

**Fig. 1:**
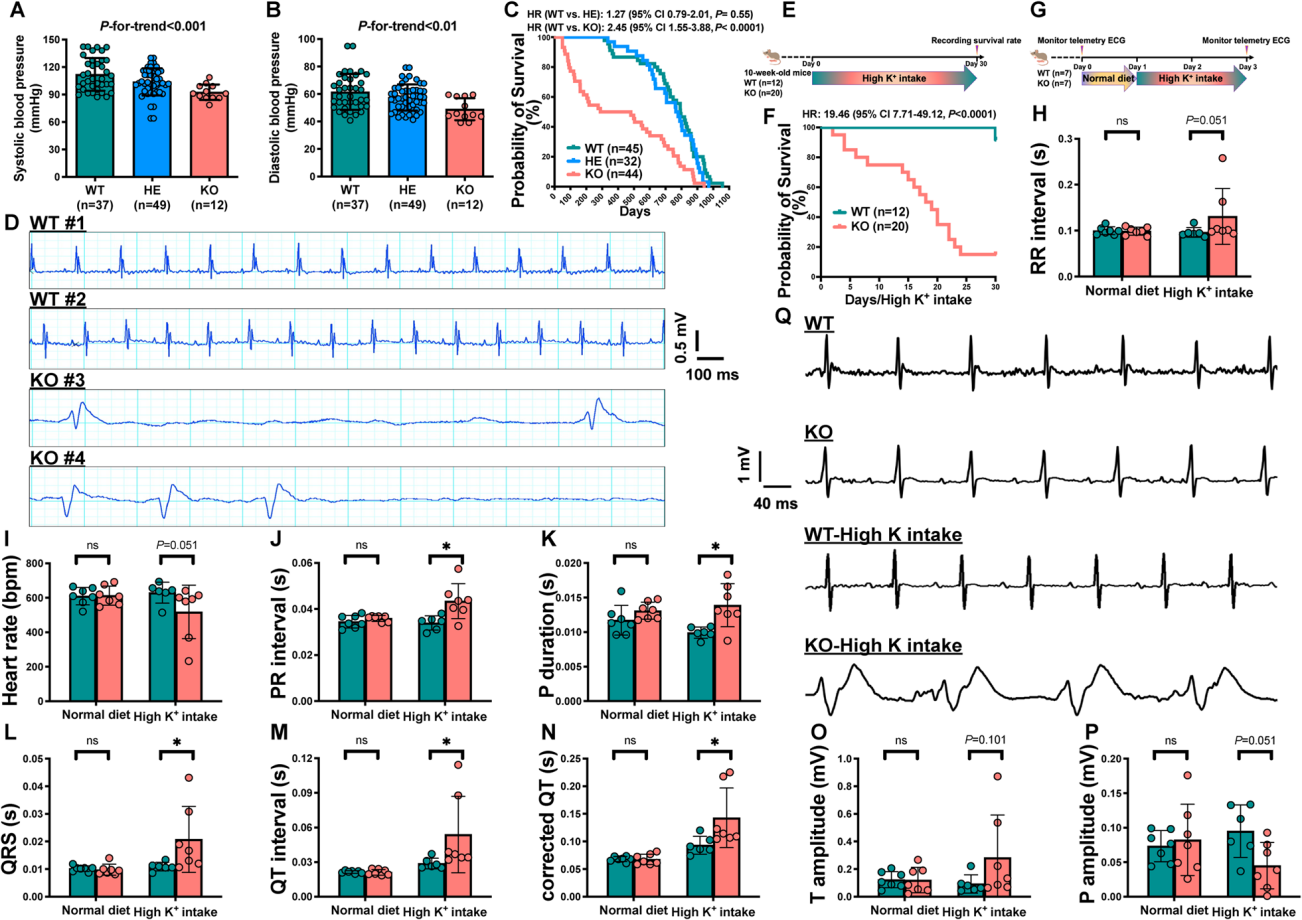
Phenotypes of *Rrbp1*-KO mice. **A** Mean value of systolic blood pressure and **B** diastolic blood pressure in mice. Data represent mean ± SEM, n = 12–49 per group. **C** Kaplan–Meier and log-rank survival analysis of *Rrbp1*-WT, *Rrbp1*-HE and *Rrbp1*-KO mice. **D** Representative electrocardiogram (ECG) of *Rrbp1*-KO and *Rrbp1*-WT mice. The ECG waveform of #1 and #2 *Rrbp1*-WT mice and the ECG waveform of terminal rhythms from #3 and #4 *Rrbp1*-KO mice. **E** Protocol for assessing the survival rate of mice under high K^+^ intake for 30 days. **F** Kaplan–Meier survival curve and log-rank survival analysis of *Rrbp1*-WT and *Rrbp1*-KO mice under high K^+^ intake for 30 days. **G** Protocol for telemetry ECG recording. **H–P** Quantitation of telemetry electrocardiogram (ECG) parameters in *Rrbp1*-KO and *Rrbp1*-WTmice with or without high K^+^ intake. RR interval; heart rate; PR interval; P duration; QRS duration; QT interval; correct QT interval; T amplitude; P amplitude (n = 6–7 per group). Representative telemetric ECG waveform of mice two days before and after high K.^+^ intake of *Rrbp1*-WT and *Rrbp1*-KO mice (**Q**). WT, wild-type; KO, knock-out. Data in (**A**) and (**B**) were analyzed with ordinary one-way ANOVA; data in (**H**)–(**P**) were analyzed with Mann–Whitney test. Data are represented as mean ± SEM. ns, no significance; *P < 0.05

*Rrbp1*-WT, *Rrbp1*-heterozygous knockout (HE), and *Rrbp1*-KO mice had systolic pressure of 112.00 ± 2.94, 103.80 ± 2.12, and 92.60 ± 2.42 mmHg, respectively (*P*-for-trend < 1 × 10^−3^) (Fig. 1A). The three groups' diastolic blood pressures were 61.53 ± 2.16, 57.53 ± 1.36, and 48.94 ± 2.31 mmHg, respectively (*P*-for-trend < 0.01) (Fig. 1B). Furthermore, *Rrbp1*-KO mice were prone to sudden death. Figure 1C shows the Kaplan–Meier survival curves of *Rrbp1*-WT, *Rrbp1*-HE, and *Rrbp1*-KO mice. *Rrbp1*-KO mice died at a higher rate than *Rrbp1*-WT mice (hazard ratio [HR]: 2.45, 95% confidence interval [CI]: 1.55–3.88, *P* < 1 × 10^−4^) (Fig. 1C). The median survival time was 481 days for *Rrbp1*-KO mice, 772 days for *Rrbp1*-HE mice, and 793 days for *Rrbp1*-WT mice. The cardiac rhythms of moribund *Rrbp1*-KO and *Rrbp1*-WT mice were monitored using surface ECG to further investigate the causes of sudden death in *Rrbp1*-KO mice. The terminal ECG of *Rrbp1*-KO mice (Fig. 1D) was consistent with severe hyperkalemia, as shown by flattened P waves, widened QRS complexes, and a tall T wave, followed by asystole [27]. The ECGs of *Rrbp1*-WT mice of the same age are shown as references.

### High K^+^ intake drastically increases sudden death in ***Rrbp1***-KO mice

*Rrbp1*-KO mice were given a high-K^+^ diet and water for a month to further investigate the causes of the high sudden death rate (Fig. 1E); they were later found to be markedly prone to sudden death (Fig. 1F). The cardiac rhythms of 12–16-week-old mice were monitored by telemetry ECG with and without high K^+^ intake to determine if the death of *Rrbp1*-KO mice under high K^+^ intake was related to life-threatening arrhythmia (Fig. 1G). Under normal dietary conditions, ECGs revealed no differences in the RR interval, heart rate, PR interval, P duration, QRS interval, QT interval, corrected QT interval, T amplitude, and P amplitude (Fig. 1H–P). However, *Rrbp1*-KO mice showed longer PR intervals, P durations, QRS intervals, QT intervals, and corrected QT intervals than*Rrbp1*-WT mice (0.044 ± 0.008 versus 0.034 ± 0.003 s; *P* < 0.05; 0.014 ± 0.003 versus 0.010 ± 0.001 secs; *P* < 0.05; 0.017 ± 0.007 versus 0.011 ± 0.001 secs; *P* < 0.05; 0.036 ± 0.005 versus 0.029 ± 0.005 secs; *P* < 0.05; 0.105 ± 0.014 versus 0.093 ± 0.016 secs; *P* < 0.05, respectively) (Fig. 1J–N) at 2 days after high K^+^ intake. There was no difference in the RR interval, heart rate, T amplitude, and P amplitude of *Rrbp1*-KO and WT mice after two days of high K^+^ intake, but the *P* values still showed a similar trend. Compared to *Rrbp1*-WT mice, the T waves of two *Rrbp1*-KO mice in the high K^+^ intake group peaked, which indicates severe hyperkalemia. Figure 1Q shows representative ECG waveforms.

### *Rrbp1*-KO mice feature volume depletion and hyporeninemic hypoaldosteronism

After the normal diet, the cardiac output of *Rrbp1*-KO mice was lower than that of *Rrbp1*-WT mice according to echocardiography. (12.99 ± 1.70 versus 15.63 ± 1.83 ml/min; *P* = 0.01) (Additional file 1: Fig. S3A). Correspondingly, the stroke volume and left ventricular volume in diastole of *Rrbp1*-KO mice were also lower than those of *Rrbp1*-WT mice (43.65 ± 5.55 versus 59.28 ± 11.01 μl; *P* < 0.01; and 69.66 ± 8.51 versus 92.26 ± 21.56 μl, respectively; *P* = 0.02) (Additional file 1: Fig. S3B, S3C). There were no significant differences in left ventricular mass, left ventricular posterior wall thickness, interventricular septum thickness in diastole, relative wall thickness, left ventricular fractional shortening, or left ventricular ejection fraction (Additional file 1: Fig. S3D-3I). Cardiac output and stroke volume were decreased in *Rrbp1*-KO mice, while their wall thickness and contractility did not change.

**Fig. 2:**
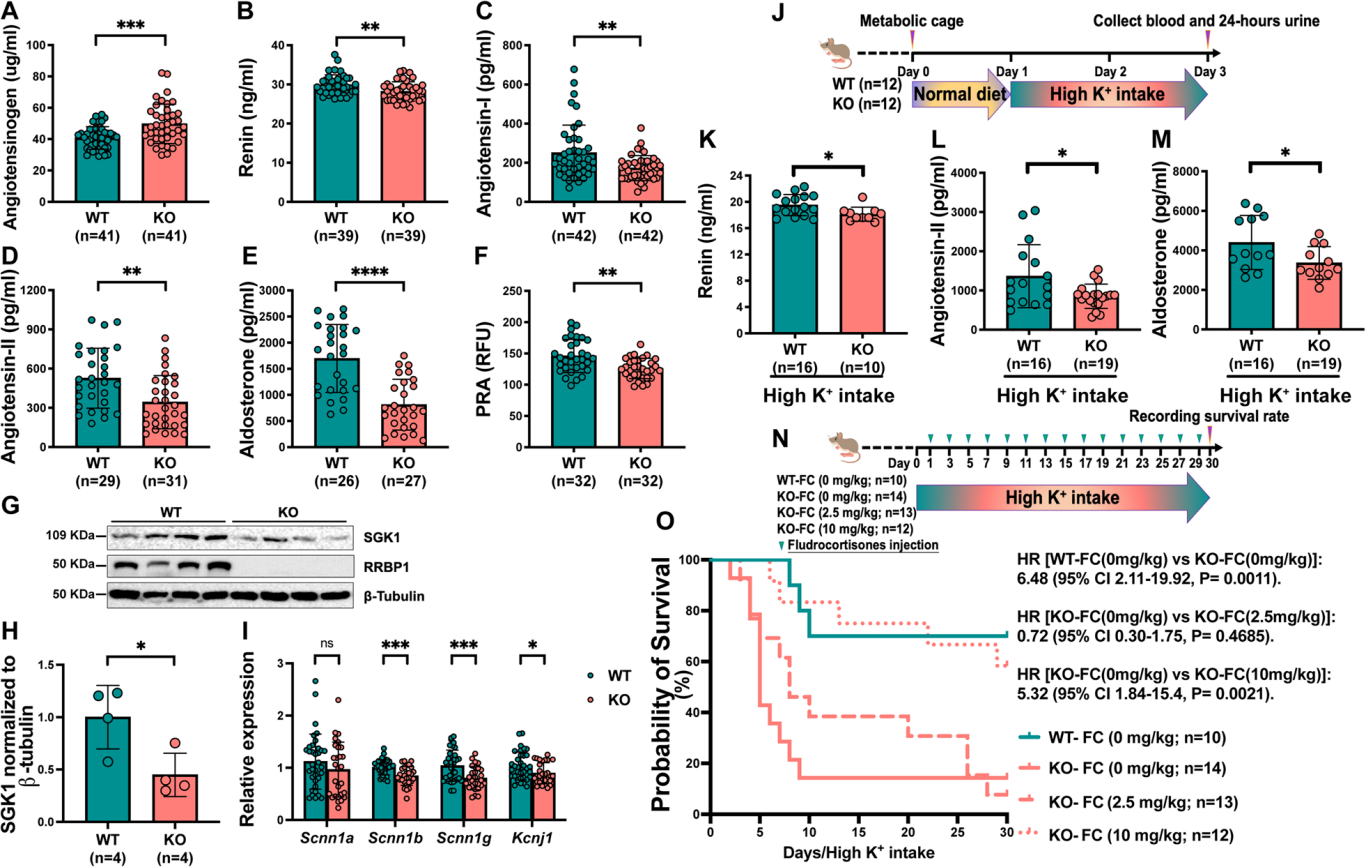
*Rrbp1*-KO mice show hyporeninemic hypoaldosteronism. **A**–**F** Plasma angiotensinogen, renin, Ang-I, Ang-II, aldosterone levels, and plasma renin activity (PRA) in mice. **G** Western blot analysis of SGK1 protein expression in kidneys harvested from *Rrbp1*-WT and *Rrbp1*-KO mice. **H** Quantification of the immunoblot in (**G**). **I** mRNA levels of *Scnn1a*, *Scnn1b*, *Scnn1g*, and *Kcnj1* in mice kidney measured using quantitative RT-PCR. Data were analyzed using the 2-ΔΔCt method with GAPDH as the reference gene (n = 28–35 per group). **J** Protocol for blood and urine test for mice that underwent high K^+^ intake. **K**–**M** Plasma renin, Ang-II, and aldosterone levels in mice that underwent high K^+^ intake for 48 h. **N** Study protocol for recording survival rate of mice that underwent high K.^+^ intake for 30 days with 0, 2.5, 10 mg/kg fludrocortisone treatment (FC) and control mice, respectively. **O** Kaplan–Meier survival curve and log-rank analysis of *Rrbp1*-KO mice rescued with 0, 2.5, 10 mg/kg fludrocortisone treatment (FC) and control mice, respectively. WT, wild-type; KO, knock-out. Data in **A**–**F**, **I**, and **K**–**M** were analyzed with an unpaired, two-tailed Student’s t-test; data in **H** were analyzed using the Mann–Whitney test. Data are represented as mean ± SEM. PRA, plasma renin activity; RFU, relative fluorescence units; ns, no significance; *P < 0.05; ** P < 0.01; ***P < 0.001; **** P < 0.0001

Plasma renin, angiotensinogen, angiotensin-I, angiotensin-II, and aldosterone were measured to assess the role of the RAAS axis in volume-related lower blood pressure and hyperkalemia. *Rrbp1*-KO mice had significantly higher plasma angiotensinogen levels than *Rrbp1*-WT mice (49.72 ± 12.53 versus 40.72 ± 7.20 µg/ml;* P* = 1 × 10^−4^) (Fig. 2A) but lower plasma renin (28.21 ± 2.47 versus 29.84 ± 2.60 ng/ml;* P* = 5.9 × 10^−3^) (Fig. 2B), angiotensin-I (172.7 ± 63.5 versus 251.0 ± 141.4 pg/ml;* P* = 1.6 × 10^−3^) (Fig. 2C), angiotensin-II (343.2 ± 203.8 versus 526.9 ± 229.5 pg/ml; *P* = 1.7 × 10^−3^) (Fig. 2D), and aldosterone (812.1 ± 486.1 versus 1697.0 ± 651.2 pg/ml;* P* < 1 × 10^−4^) (Fig. 2E).The plasma renin activity (PRA) was also significantly lower in knockout mice (Fig. 2F) (*P* < 0.01). The expression of serum/glucocorticoid regulated kinase 1 (SGK1) as an early aldosterone-induced protein significantly decreased in kidney homogenates from *Rrbp1*-KO mice (Fig. 2G, H); SGK1 is an aldosterone-responsive protein that modulates the expression and function of various renal ion channels such as epithelial Na^+^ channel ENaC and renal K^+^ channel ROMK to regulate sodium reabsorption and K^+^ secretion [28]. RT-qPCR revealed no difference in levels of *Scnn1a* encoding ENaC-α protein expression, but lower levels of *Scnn1b*, *Scnn1g*, and *Kcnj1* encoding ENaC-β, ENaC-γ, and ROMK were found in *Rrbp1*-KO mice compared to wild-type controls (Fig. 2I). H&E staining, adrenal gland weight, and ACTH (adrenocorticotropic hormone) stimulation test were performed to characterize the adrenal gland in *Rrbp1*-KO mice (Additional file 1: Fig. S4). There were no obvious lesions or weight changes in adrenal glands from *Rrbp1*-WT *and Rrbp1*-KO mice. The basal plasma level of corticosterone was lower in *Rrbp1*-KO mice, suggesting that the lower plasma angiotensin-II level in *Rrbp1*-KO reduced corticosterone levels in plasma. After ACTH stimulation, there was no significant difference in plasma levels of corticosterone in *Rrbp1*-WT *and Rrbp1*-KO mice. The basal plasma potassium level was higher in *Rrbp1*-KO mice compared with *Rrbp1*-WT mice (6.77 ± 0.14 versus 6.28 ± 0.11 mM; *P* < 0.01). The basal plasma potassium value was approximately 6.28 mM in *Rrbp1*-WT mice rather than 4–5 mM in normal C57BL/6 mice, which could be attributed to the mixed genetic background of the C57BL6/129J mice. In addition, the basal fractional excretion of potassium was significantly lower in *Rrbp1*-KO mice compared to *Rrbp1*-WT mice (11.52 ± 0.843 versus 16.44 ± 0.997; *P* < 1 × 10^−3^) (Table 2). Moreover, *Rrbp1*-KO mice still had lower plasma renin (18.12 ± 1.08 versus 19.51 ± 1.601 ng/ml;* P* = 0.02) (Fig. 2K), angiotensin-II (855.7 ± 310.8 versus 1365 ± 803.5 pg/ml;* P* = 0.02) (Fig. 2L), and aldosterone (3361 ± 826.2 versus 4397 ± 1370 pg/ml;* P* = 0.04) levels than *Rrbp1*-WT mice (Fig. 2M) after 2 days of high K^+^ intake (Fig. 2J). Consistently, plasma K^+^ concentration significantly increased in *Rrbp1*-KO mice compared to *Rrbp1*-WT mice (6.77 ± 0.14 versus 6.28 ± 0.11 mmol/L;* P* < 0.01). Two days after high K^+^ intake, *Rrbp1*-KO mice had higher serum potassium levels (8.53 ± 0.38 versus 7.23 ± 0.27 mmol/L;* P* = 0.0086), decreased transtubular potassium gradient (TTKG) (14.62 ± 0.79 versus 18.44 ± 1.04;* P* = 5.8 × 10^−3^), and lower urine fractional excretion of potassium (43.14 ± 4% versus 64.84 ± 6.7%;* P* = 8.1 × 10^−3^) than *Rrbp1*-WT mice (Table 3), indicating that *Rrbp1*-KO mice developed hyporeninemic hypoaldosteronism with hyperkalemia.

**Table 2:** Plasma and urine electrolyte levels in *Rrbp1*-WT and *Rrbp1*-KO mice

Genotype (n)	*Rrbp1*-WT (n = 21)	*Rrbp1*-KO (n = 16)
*Blood*		
[K^+^], mmol/L	6.28 ± 0.11	6.77 ± 0.14 †
[Na^+^], mmol/L	149.6 ± 0.65	149.6 ± 0.71
[Cl^−^], mmol/L	113.9 ± 0.59	113.9 ± 0.59
*Urine*		
TTKG	9.5 ± 0.1	9.1 ± 0.2
FE_K_^+^	16.4 ± 1.0	11.5 ± 0.8 ‡
FE_Na_^+^	0.32 ± 0.02	0.24 ± 0.02 *
FE_Cl_^−^	0.70 ± 0.04	0.56 ± 0.05 *

TTKG, transtubular potassium gradient; FE, fractional excretion

*P < 0.05, †*P* < 0.01, ‡*P* < 0.001 *Rrbp1*-KO (knock-out) vs. *Rrbp1*-WT (wild-type)

**Table 3:** Plasma and urine electrolyte levels in *Rrbp1*-WT and *Rrbp1*-KO mice under high K^+^ intake

Genotype (n)	*Rrbp1*-WT (n = 21)	*Rrbp1*-KO (n = 16)
*Blood*		
[K^+^], mmol/L	7.23 ± 0.27	8.53 ± 0.38 †
[Na^+^], mmol/L	157.7 ± 1.32	155.9 ± 1.62
[Cl^−^], mmol/L	122.6 ± 1.61	123.4 ± 2.03
*Urine*		
TTKG	18.4 ± 1.0	14.6 ± 0.8 †
FE_K_^+^	64.8 ± 6.7	43.1 ± 4.0 ‡
FE_Na_^+^	0.42 ± 0.05	0.42 ± 0.04
FE_Cl_^−^	3.24 ± 0.33	2.63 ± 0.27

TTKG, transtubular potassium gradient; FE, fractional excretion

^†^*P* < 0.01, ‡*P* < 0.001 *Rrbp1*-KO (knock-out) vs. *Rrbp1*-WT (wild-type)

### Fludrocortisone rescues high K^+^ load-induced sudden death in ***Rrbp1***-KO mice

To confirm the relationship between high K^+^ load-induced sudden death of *Rrbp1*-KO mice and suppression of the RAAS system, mice were rescued by fludrocortisone, a synthetic mineralocorticoid (0, 2.5, 10 mg/kg intraperitoneal injection once every two days) for 30 days (Fig. 2N). Fludrocortisone improved the survival of *Rrbp1*-KO mice dose-dependently (Fig. 2O). Accordingly, there were no significant differences in levels of blood Na^+^, K^+^, Cl^−^ or excretion of urinary potassium measured two days after high K^+^ intake with or without 100 mg/kg fludrocortisone treatment (Table 4).

**Table 4:** Plasma and urine electrolyte levels in *Rrbp1*-WT and *Rrbp1*-KO mice under high K^+^ intake for 48 h with and without fludrocortisone treatment

Genotype + treatment (n)	*Rrbp1*-WT + saline (n = 22)	*Rrbp1*-KO + saline (n = 27)	*Rrbp1*-KO + Fludrocortisone (n = 18)
*Blood*			
[K^+^], mmol/L	7.49 ± 0.10	8.19 ± 0.14 ‡	7.73 ± 0.16
[Na^+^], mmol/L	161.5 ± 1.10	163.6 ± 0.72	161.6 ± 0.88
[Cl^−^], mmol/L	124.6 ± 0.99	127.1 ± 0.77	124.6 ± 1.35
*Urine*			
TTKG	29.9 ± 1.1	30.2 ± 1.3	30.6 ± 1.6
FE_K_^+^	25.3 ± 3.4	27.8 ± 2.9	20.9 ± 3.6
FE_Na_^+^	0.62 ± 0.09	0.65 ± 0.06	0.48 ± 0.07
FE_Cl_^−^	1.15 ± 0.17	1.39 ± 0.15	0.98 ± 0.15

TTKG, transtubular potassium gradient; FE, fractional excretion

^‡^*P* < 0.001 *Rrbp1*-KO (knock-out) vs. *Rrbp1*-WT (wild-type)

### Deficiency of RRBP1 increases intracellular renin level and decreases renin secretion in vivo and *vitro*

**Fig. 3:**
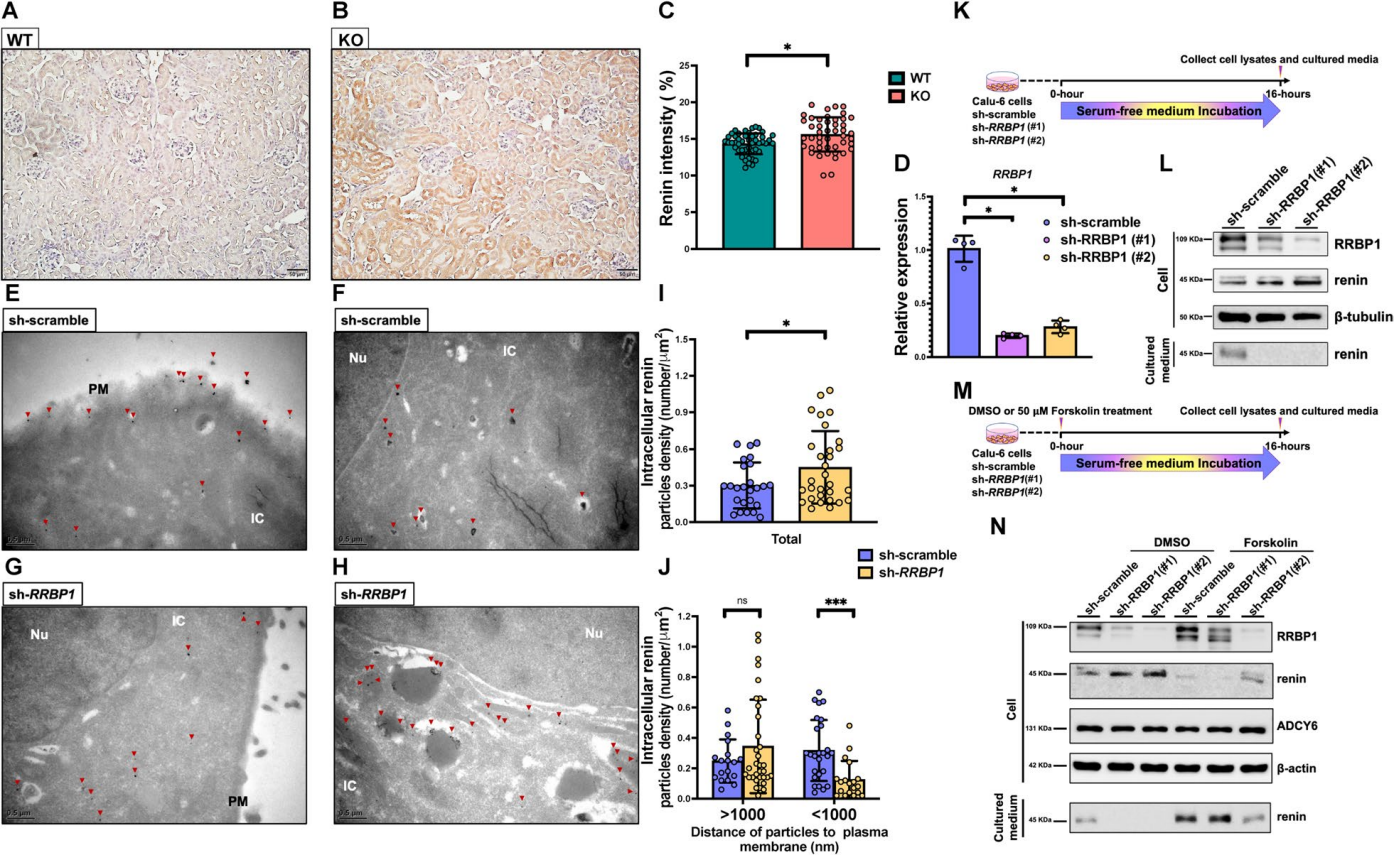
*RRBP1* deficiency decreases renin transportation and secretion. **A**,** B** Representative renin immunohistochemical staining of kidneys of *Rrbp1*-WT and *Rrbp1*-KO mice. **C** Quantification of stain intensity in kidneys from *Rrbp1*-WT and *Rrbp1*-KO mice (scale bar = 20 μm). **D** Intracellular mRNA levels of *RRBP1* of scramble-control and sh-*RRBP1* knockdown Calu-6 cells were measured using quantitative RT-PCR. Data were analyzed using the 2-ΔΔCt method with GAPDH as the reference gene (n = 4 per group). **E**–**H** Representative immunogold staining of renin by transmission electron microscopy (TEM) in control and *RRBP1* knockdown Calu-6 cells. Dark red arrow indicates the nanogold particle (scale bar = 0.5 μm). Nu, nucleus; IC, intracellular; PM, plasma membrane. **I** Quantification of intracellular renin particles by TEM in control and *Rrbp1* knockdown Calu-6 cells. **J** Quantification of intracellular renin particles with a distance of more and less than 1000 nm from plasma membranes. **K** Protocol for collecting cell lysates and supernatant of control and *RRBP1* knockdown Calu-6 cells. **L** Western blot analysis of RRBP1, renin protein expression in *RRBP1*-knockdown Calu-6 cells, and supernatant. **M** Protocol for forskolin-induced renin production in control and *RRBP1* knockdown cells. **N** Western blot analysis of RRBP1, renin, and ADCY6 protein expression in *RRBP1*-knockdown Calu-6 cells and supernatant. Lanes 1–3 represent cells with DMSO control treatment. Lane 4–6 represents cells induced with 50 μM forskolin. WT, wild-type; KO, knock-out. Data in **C**, **E**, **J** were analyzed with an unpaired, two-tailed Student’s t-test; data in (**D**) were analyzed using a Mann–Whitney test. Data were represented as mean ± SEM. ns, no significance; *P < 0.05; ***P < 0.001

Immunostaining was used to measure renin expression in the kidneys of *Rrbp1*-KO and *Rrbp1*-WT mice because *Rrbp1*-KO mice showed low PRA (Fig. 3A, B). Unexpectedly, the intensity of renin stain was significantly higher in *Rrbp1*-KO than in *Rrbp1-*WT mice (15.35 ± 2.93 versus 14.51 ± 1.62 arbitrary units;* P* < 0.05) in the kidneys (Fig. 3C).

To clarify how RRBP1 affects renin distribution, *RRBP1* was knocked down in cultured Calu-6 cells (Fig. 3D), a human renin-producing cell line, which were stained with immunogold-labeled anti-renin antibody and examined by transmission electron microscopy (TEM) (Fig. 3E–H). The total intracellular renin particles increased in *RRBP1*-knockdown Calu-6 cells relative to scramble controls (*P* < 0.05) (Fig. 3I). TEM data showed that renin particles were more distant from the plasma membrane (> 1000 nm) in *RRBP1*-knockdown cells compared to controls (Fig. 3J). These results indicated that more renin particles accumulated intracellularly, and less renin was transported to the plasma membrane and secreted by *RRBP1*-knockdown cells.

Next, renin levels were measured in *RRBP1*-knockdown cells to explore how RRBP1 affects renin secretion. Indeed, less renin was secreted into the culture medium of *RRBP1*-knockdown cells, whereas more renin accumulated in those cells (Fig. 3K, L).

Renin secretion is controlled by the cyclic adenosine monophosphate (cAMP) signaling in response to various external stimuli [10]. However, the expression level of adenylyl cyclase 6 (*ADCY6*), the major enzyme responsible for producing intracellular cAMP [10, 29], was not significantly different in Calu-6 cells (Additional file 1: Fig. S5A).

Cells were induced with forskolin, an adenylyl cyclase activator, to enhance intracellular cAMP levels and therefore test if enhanced intracellular cAMP levels can rescue renin secretion during *RRBP1* deficiency. After forskolin induction, intracellular cAMP levels became comparable to those of controls (Additional file 1: Fig. S5B). Correspondingly, forskolin induction reversed the decreased renin secretion into the culture medium and the accumulation of renin within *RRBP1*-knockdown cells (Fig. 3M and N). However, the level of secreted renin detected in the culture medium was still lower in *RRBP1*-knockdown cells #2 compared to those that of control cells and *RRBP1*-knockdown cells #1, which may be attributed to the significantly lower knockdown efficiency of knockdown cells #1 compared with #2 in Fig. 3N after forskolin induction. Additionally, the level of secreted renin in sh-*RRBP1*#1 cells with very low knockdown efficiency was comparable to that of control cells after forskolin treatment (Fig. 3N). Despite the difference in knockdown efficiency, these findings, however, indicate that renin secretion is strongly related to RRBP1 expression. These findings suggest that renin secretion is strongly related to RRBP1 expression; RRBP1 deficiency still reduces renin secretion despite increased intracellular cAMP.

### Deficiency of RRBP1 causes retention of renin in the endoplasmic reticulum

**Fig. 4:**
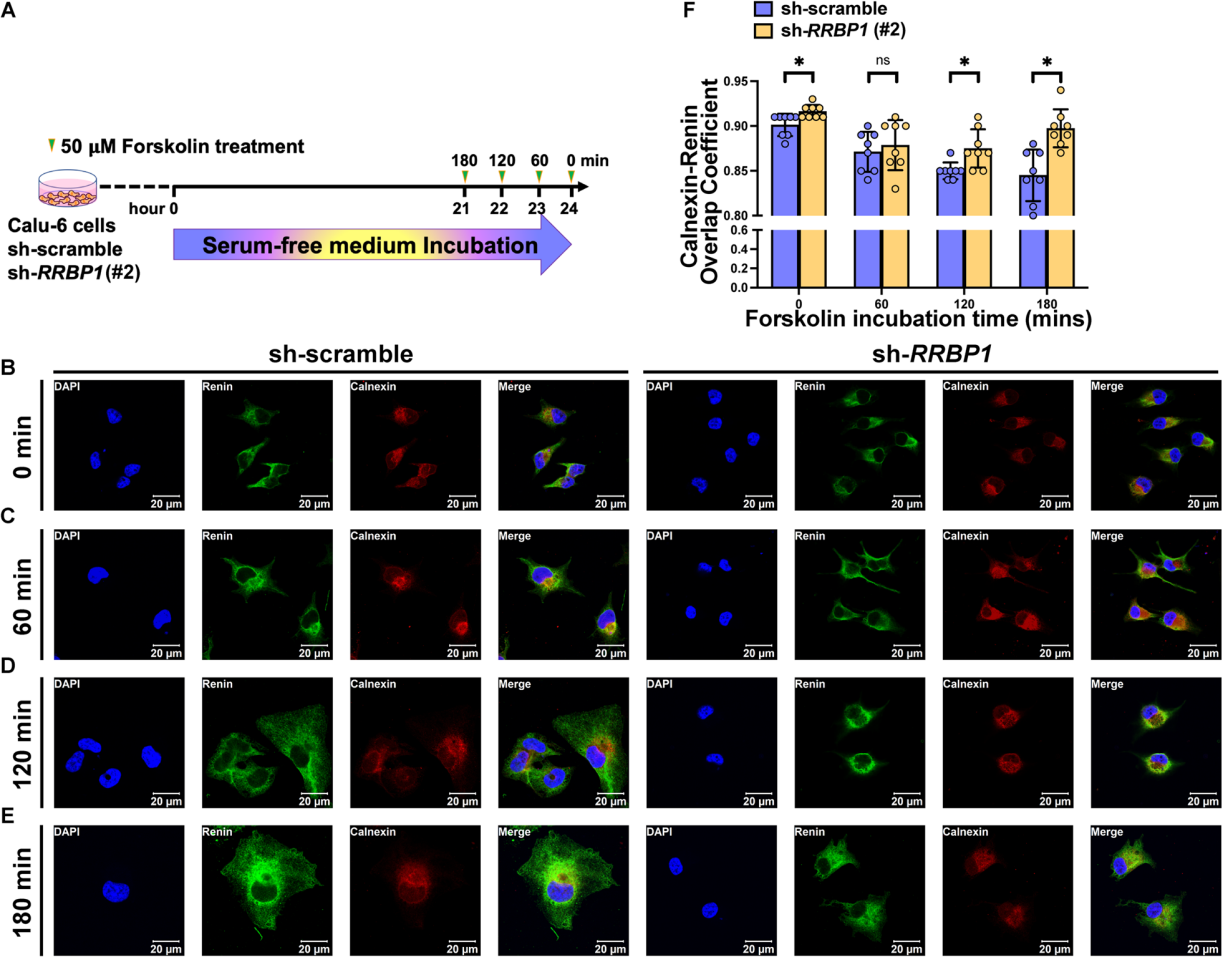
RRBP1 deficiency increases renin accumulation in ER. **A** Protocol to stimulate renin trafficking in control and *RRBP1* knockdown cells. **B**–**E** Representative confocal microscopy images of control and *RRBP1* knockdown Calu-6 cells showing renin (green), calnexin (red), and DAPI (blue) after 50 µM of forskolin induction for 0, 60, 120, and 180 min. **F** Overlap coefficients of renin (green) and calnexin (red) in control and *RRBP1* knockdown Calu-6 cells after 50 µM of forskolin induction for 0, 60, 120, and 180 min (n = 8 per group). Data in (**F**) were analyzed with an unpaired, two-tailed Student’s t-test. Data are represented as mean ± SEM. ns, no significance; *P < 0.05

**Fig. 5:**
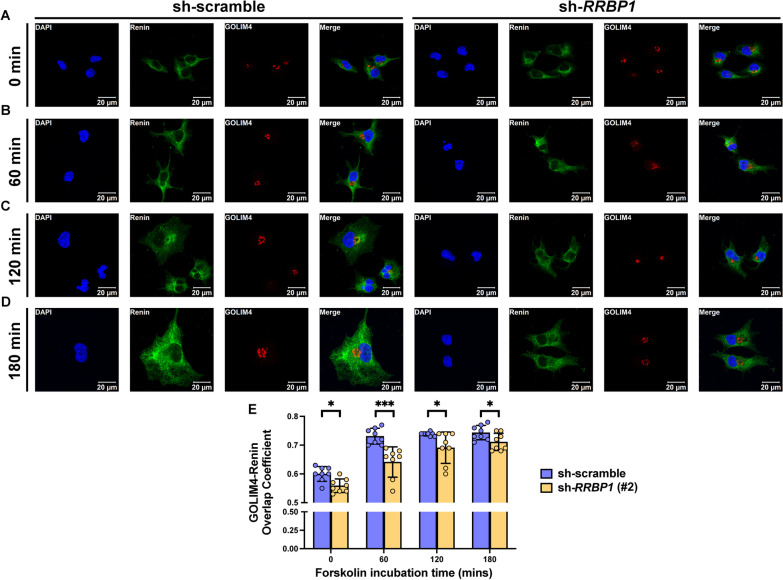
RRBP1 deficiency reduces renin transport from ER to Golgi apparatus. **A**–**D** Representative confocal microscopy images of control and *RRBP1* knockdown Calu-6 cells showing renin (green), GOLIM4 (red), and DAPI (blue) after 50 µM of forskolin induction for 0, 60, 120, and 180 min. **E** Overlap coefficients of renin (green) and GOLIM4 (red) in control and *RRBP1* knockdown of Calu-6 cells after 50 µM of forskolin induction for 0, 60, 120, and 180 min (n = 8 per group). Data in (**E**) were analyzed with an unpaired, two-tailed Student’s t-test. Data are represented as mean ± SEM. *P < 0.05; ***P < 0.001

**Fig. 6:**
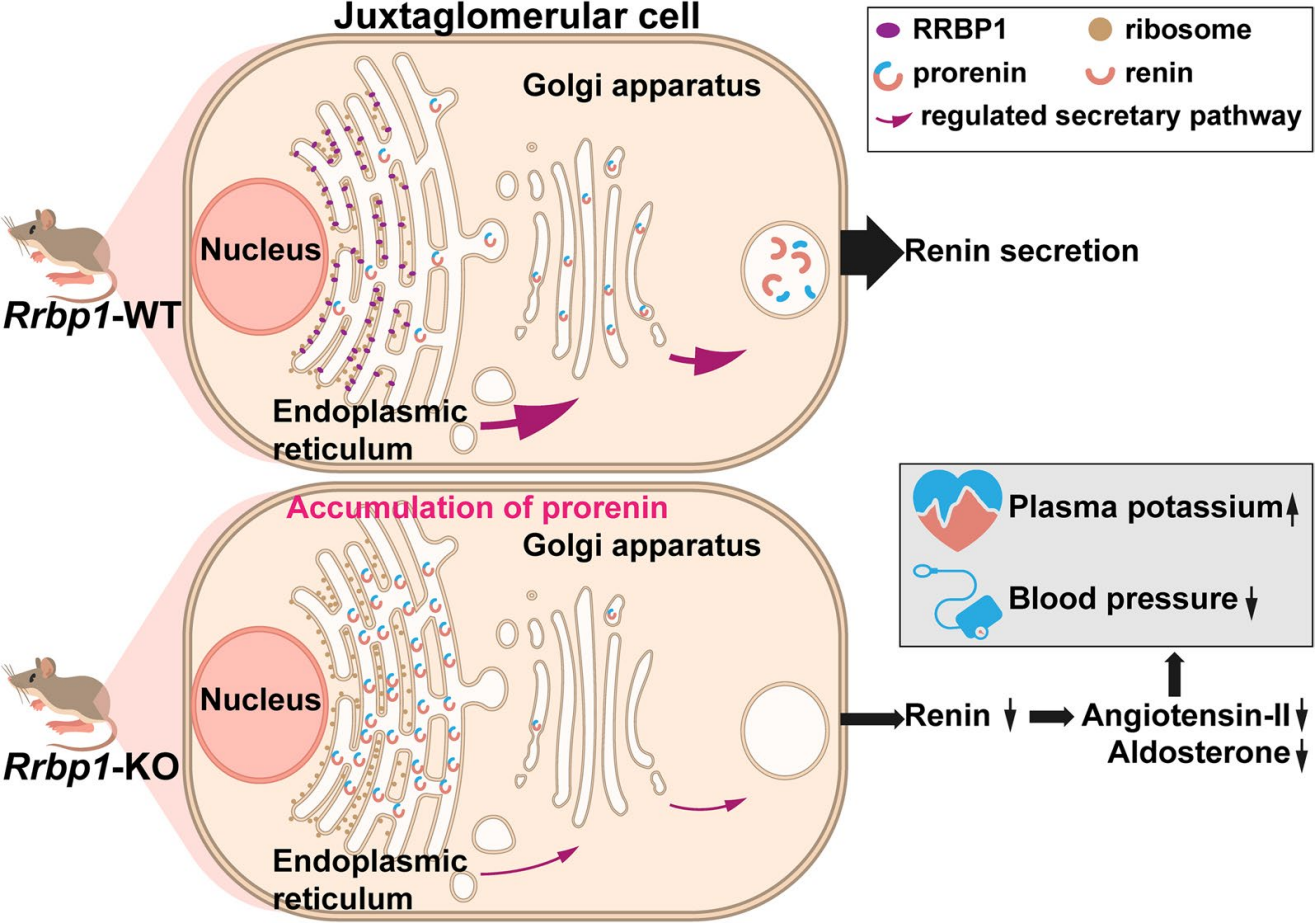
Schematic of the mechanism through which RRBP1 modulates renin trafficking and secretion. RRBP1 deficiency causes hyporeninemic hypoaldosteronism, lower blood pressure, and hyperkalemia

After forskolin induction, cells were immune-stained with anti-renin, anti-RRBP1, anti-calnexin (an ER marker), and anti-GOLIM4 (a Golgi apparatus marker) to investigate intracellular trafficking of renin in *RRBP1*-knockdown and control cells (Fig. 4A and Additional file 1: Fig. S6). At 0, 60, 120, and 180 min post-induction, confocal microscopy revealed that renin was retained in the ER (Fig. 4B–E) and not transported to the Golgi apparatus (Fig. 5A-D) upon forskolin induction in *RRBP1*-knockdown Calu-6 cells. However, renin was transported to the Golgi apparatus upon forskolin induction in scramble controls. To quantify the intracellular trafficking of renin, we calculated the fluorescent signal intensity of renin overlapped with calnexin as well as with GOLIM4. Initially, the overlapped signal of renin with calnexin did not differ between *RRBP1*-knockdown cells and controls. However, after forskolin induction for 120 and 180 min, the overlapped fluorescent signal between renin and calnexin of *RRBP1*-knockdown cells was higher than that of control cells (0.875 ± 0.021 versus 0.850 ± 0.009;* P* = 0.02) and (0.898 ± 0.021 versus 0.845 ± 0.029;* P* = 1 × 10^−3^) (Fig. 4F). On the contrary, the overlapped fluorescent signal between renin and GOLIM4 of *RRBP1*-knockdown cells was consistently lower compared to that of control cells after forskolin induction at 0 (0.559 ± 0.024 versus 0.600 ± 0.026;* P* = 8 × 10^−3^), 60 (0.641 ± 0.053 versus 0.731 ± 0.027;* P* < 1 × 10^−3^), 120 (0.691 ± 0.055 versus 0.739 ± 0.006;* P* = 0.04), and 180 (0.711 ± 0.030 versus 0.744 ± 0.025;* P* = 0.03) minutes, respectively (Fig. 5E). These findings indicated that the renin was retained in the ER of *RRBP1*-knockdown cells. Accordingly, renin required more time to leave the ER and enter the Golgi apparatus in the *RRBP1*-knockdown cells after stimulation. Ultimately, these data show that RRBP1 regulates renin trafficking between the ER and the Golgi apparatus as well as renin secretion. RRBP1 deficiency causes hyporeninemic hypoaldosteronism and hyperkalemia (Fig. 6).

## Discussion

This study associated *RRBP1* genetic variants with blood pressure in a family-based genome-wide linkage and regional fine mapping of a SAPPHIRe cohort. Transgenic mice experiments showed that RRBP1 deficiency caused volume-related lower blood pressure and severe hyperkalemia-induced sudden cardiac death. *Rrbp1*-KO mice displayed features typical of hyporeninemic hypoaldosteronism with hyperkalemia, which could be ameliorated by fludrocortisones. Knockdown of *RRBP1* in renin-producing cell lines resulted in accumulation of renin in ER and subsequently reduced renin secretion. This is the first study to show that RRBP1 regulates the RAAS axis by modulating intracellular trafficking and secretion of renin.

Previous GWAS have linked *RRBP1* genetic variants to CVDs, including coronary artery disease and intracranial arterial aneurysm [1–6]. *RRBP1* genetic variants have also been associated with serum apolipoprotein B, apolipoprotein A1, low-density lipoprotein cholesterol, high-density lipoprotein cholesterol, and total cholesterol levels [8, 30, 31]. According to one study, RRBP1 regulates inter-organellar contact between rough ER and mitochondria to modulate the biogenesis and secretion of very-low-density lipoproteins (VLDLs) in the liver [22]. These findings suggest that RRBP1 influences CVD risk by regulating lipoprotein biosynthesis and secretion.

Additionally, we found that *RRBP1* genetic variants are associated with blood pressure. RRBP1 deficiency in mice decreased blood pressure in mice, consistent with other human genetic association studies. Of note, *Rrbp1*-KO mice increasingly experienced unexpected sudden death. Arrhythmia is the most leading cause of cardiac sudden death [32]. The terminal rhythms were compatible with arrhythmia caused by hidden hyperkalemia, without detailed pathological and structural abnormalities in the hearts, coronary arteries, aortas or whole brains (Additional file 1: Fig. S7) of *Rrbp1*-KO mice. Hyperkalemia is a “silent killer” since it can cause unexpected arrhythmia and sudden cardiac death [33, 34]. The sudden death events of *Rrbp1*-KO mice associated with hyperkalemia were furthered validated by exposing mice to a 30-day high K^+^ intake, during which the sudden death events of *Rrbp1*-KO mice increased dramatically. Generally, abnormal ECG patterns are related to serum K^+^ concentrations [35]. ECG waveforms with mild (5.5–6.5 mmol/l) and moderate (6.5–8 mmol/l) changes in serum K^+^ levels might be subtle or absent, but some still displayed peaked T waves, prolonged PR and QRS intervals, and S-T elevation in humans [36]. However, the waveform of ECGs with severe hyperkalemia (> 8 mmol/l) may display a progressively widening QRS interval, axis deviations, and asystole [36, 37]. Of note, *Rrbp1*-KO mice presented with prolonged RR, PR, QT, and correct QT intervals as well as progressive widening of the QRS complex and tall T waves during telemetry monitoring under exposure to high-K^+^ intake for two days. The serum K^+^ concentration of *Rrbp1*-KO mice reached 8.53 ± 0.38 mmol/l, which was significantly higher than that of wild-type mice after high-K^+^ loading for 2 days. Fludrocortisone treatment dose-dependently decreased sudden death rate, reiterating that severe hyperkalemia causes sudden death.

*Rrbp1*-KO mice showed hyperkalemia with reduced urine K^+^ excretion, indicating defective renal tubular K^+^ secretion; they were, however, phenotypically corrected by fludrocortisone, suggesting hypoaldosteronism rather than pseudo-hypoaldosteronism. *Rrbp1*-KO mice showed major defects in renin production and secretion, leading to hyporeninemia and hypoaldosteronism.

The plasma levels of angiotensin-II and aldosterone in both *Rrbp1*-WT and KO were increased after high K^+^ intake. However, we also found that the plasma renin levels in both *Rrbp1*-WT and KO with high K^+^ intake were lower than those of normal-diet group. Previous studies have clearly demonstrated that plasma angiotensin-II and aldosterone levels directly increased in response to elevation of plasma potassium level [38, 39]. Angiotensin-II is the major tonic regulator of renin secretion via negative feedback regulation. Angiotensin-II inhibits the release of renin via AT1 receptors on juxtaglomerular cells, lowering plasma renin activity and the production of angiotensin-I and angiotensin-II [40–42].

Although many cell types can generate prorenin, which allegedly has little enzyme activity, only JG cells in kidney can produce active renin that responds to external stimuli such as volume depletion [43, 44]. Further immunohistochemistry staining showed stronger renin intensity in kidney sections from *Rrbp1*-KO mice, contrary to the lower renin levels in plasma. These findings indicate that renin accumulates in the kidney of *Rrbp1*-KO mice rather than secreting into circulation.

To address the relationship between RRBP1 and renin production, this study used Calu-6, a human renin-producing cell line that expresses endogenous human renin and promoter activity [45]. Renin levels decreased in the culture medium of *RRBP1*-knockdown cells. On the contrary, the renin level was higher and accumulated intracellularly in *RRBP1*-knockdown cells, suggesting that *RRBP1* regulates renin maturation and secretion. TEM also showed greater intracellular retention of renin that was more distant from the plasma membrane in *RRBP1*-knockdown cells compared to controls. Immunofluorescence further showed that renin is retained in the ER and not transported to the Golgi apparatus in *RRBP1*-knockdown cells.

This study has several limitations. First, we did not consider contributions of organs or tissues in blood pressure regulation; *Rrbp1* expression is not restricted to JG cells. Further cell or tissue-specific knockout mice studies may be warranted. Moreover, the basal levels of cAMP in *RRBP1*-knockdown cells were lower than in control cells (Additional file 1: Fig. S5B), suggesting that RRBP1 regulates intracellular cAMP levels. This should be confirmed in future studies.

Clinically, the most common conditions associated with hyporeninemic hypoaldosteronism are diabetic nephropathy and other renal disorders [46–49]. Hyperkalemia is a potentially life-threatening electrolyte imbalance often caused by hyporeninemic hypoaldosteronism [48]. An inadequate release of renin or insufficient conversion of prorenin into renin are the leading causes of hyporeninemic hypoaldosteronism [47–49].

## Conclusion

The present study shows that deficiency of RRBP1 impairs the trafficking of renin from ER to the Golgi apparatus for further secretion. RRBP1-deficient mice manifested clinical features of hyporeninemic hypoaldosteronism, leading to lower blood pressure, severe hyperkalemia, and sudden cardiac death. This study identifies RRBP1 as a novel regulator of blood pressure and potassium homeostasis.

## Supplementary Information

**Additional file 1. **Tables S1–S3, Figures S1–S7, Supplemental Materials and Methods.

## Data Availability

All data are included in this published article (and its Additional file).

## Abbreviations

ADCY6: Adenylate cyclase type 6
CVD: Cardiovascular disease
FE: Fractional excretion
GOLIM4: Golgi integral membrane protein 4
GWAS: Genome-wide association study
HE: Heterozygous
Kcnj1: Potassium inwardly rectifying channel subfamily j member 1
KO: Knockout
Neo: Neomycin
QTL: Quantitative trait locus
RAAS: Renin–angiotensin–aldosterone system
SAPPHIRe: Stanford Asia–Pacific Program for Hypertension and Insulin Resistance
Scnn1a: Sodium channel epithelial 1 subunit alpha
Scnn1b: Sodium channel epithelial 1 subunit beta
Scnn1g: Sodium channel epithelial 1 subunit gamma
SGK1: Serum/glucocorticoid regulated kinase 1
TTKG: The transtubular K^+^ gradient
VLDL: Very-low-density lipoproteins
WT: Wild-type
