# Supplementary material for: ER ribosomal-binding protein 1 regulates blood pressure and potassium homeostasis by modulating intracellular renin trafficking
Source: J Biomed Sci. 2023 Feb 19;30:13. doi: 10.1186/s12929-023-00905-7 (PMC9940419; doi:10.1186/s12929-023-00905-7)
Supplement: Supplementary file 1 — Additional file 1. Tables S1–S3, Figures S1–S7, Supplemental Materials and Methods. [file 12929_2023_905_MOESM1_ESM.docx]

ER ribosomal-binding protein 1 regulates blood pressure and potassium homeostasis by modulating intracellular renin trafficking

Chu-Hsuan Chiu^1,2^, Chin-Feng Hsuan^3,4,5^, Shih-Hua Lin^6,7^, Yi-Jen Hung^8^, Chii-Min Hwu^9,10^, Siow-Wey Hee^11^, Shu-Wha Lin^12^, Sitt-Wai Fong^2^, Patrick Ching-Ho Hsieh ^2^, Wei-Shun Yang^1,13^, Wei-Chou Lin^14^, Hsiao-Lin Lee^11^, Meng-Lun Hsieh^11,15^, Wen-Yi Li^16^, Jou-Wei Lin^17^, Chih-Neng Hsu^17^, Vin-Cent Wu^18^, Gwo-Tsann Chuang^1,19^, Yi-Cheng Chang^1,2,11,20*^, Lee-Ming Chuang^11,20,21*^

*** Corresponding Authors**

* Chang Yi-Cheng

**Email:** [b83401040@gmail.com](mailto:b83401040@gmail.com)

* Chuang Lee-Ming

**Email:** leeming@ntu.edu.tw

**Table S1**. Plasma and urine test for *Rrbp1*-WT and *Rrbp1*-KO mice.

| Genotype  (n) | *Rrbp1*-WT  (n = 21) | *Rrbp1*-KO  (n = 16) |
| --- | --- | --- |
| Blood | | |
| [K^+^], mmol/L | 6.28 ± 0.11 | 6.77 ± 0.14 † |
| [Na^+^], mmol/L | 149.6 ± 0.65 | 149.6 ± 0.71 |
| [Cl^-^], mmol/L | 113.9 ± 0.59 | 113.9 ± 0.59 |
| BUN, mg/dL | 20.77 ± 0.67 | 20.62 ± 1.05 |
| Cre, mg/dL | 0.23 ± 0.01 | 0.18 ± 0.09 |
| Glu, mmol/L | 141.8 ± 6.20 | 145.8 ± 6.15 |
| Urine | | |
| Volume, ml | 1.05 ± 0.20 | 1.12 ± 0.16 |
| K^+^, µmol | 321 ± 31.44 | 397.2 ± 43.08 |
| Na^+^, µmol | 153.3 ± 20.20 | 188.2 ± 26.32 |
| Cl^-^, µmol | 256.8 ± 29.50 | 314.5 ± 33.50 |
| BUN, mg | 41.42 ± 4.54 | 52.28 ± 5.67 |
| Cre, mg | 0.71 ± 0.07 | 0.88 ± 0.10 |
| Glu, mg | 0.75 ± 0.13 | 0.64 ± 0.08 |
| BUN, Blood urea nitrogen; Cre, Creatinine; Glu, Glucose; †*P* < 0.01, *Rrbp1*-KO (knock-out) vs. *Rrbp1*-WT (wild-type) | | |

**Table S2.** Plasma and urine test for *Rrbp1*-WT and *Rrbp1*-KO mice under high K^+^ intake.

| Genotype  (n) | *Rrbp1*-WT  (n = 21) | *Rrbp1*-KO  (n = 16) |
| --- | --- | --- |
| Blood | | |
| [K^+^], mmol/L | 7.23 ± 0.27 | 8.53 ± 0.38† |
| [Na^+^], mmol/L | 157.7 ± 1.32 | 155.9 ± 1.62 |
| [Cl^-^], mmol/L | 122.6 ± 1.61 | 123.4 ± 2.03 |
| BUN, mg/dL | 30.29 ± 1.30 | 27.2 ± 1.07 |
| Cre, mg/dL | 0.25 ± 0.02 | 0.20 ± 0.01 |
| Glu, mmol/L | 124.1 ± 4.79 | 113.3 ± 4.95 |
| Urine | | |
| Volume, ml | 1.25 ± 0.14 | 1.69 ± 0.08† |
| K^+^, µmol | 1053 ± 116.90 | 1140 ± 75.95 |
| Na^+^, µmol | 136.1 ± 10.73 | 204.0 ± 12.89‡ |
| Cl^-^, µmol | 912.3 ± 105.30 | 1011.5 ± 72.07 |
| BUN, mg | 32.04 ± 2.10 | 40.14 ± 2.25 |
| Cre, mg | 0.52 ± 0.04 | 0.64 ± 0.03 |
| Glu, mg | 0.80 ± 0.09 | 1.16 ± 0.17 |
| BUN, Blood urea nitrogen; Cre, Creatinine; Glu, Glucose; †*P* < 0.01, ‡*P* < 0.001*Rrbp1*-KO (knock-out) vs. *Rrbp1*-WT (wild-type) | | |

**Table S3**. Plasma and urine test for *Rrbp1*-WT and *Rrbp1*-KO mice under high K^+^ intake for 48 hours with and without fludrocortisone treatment.

| Genotype  +  treatment  (n) | *Rrbp1*-WT  +  saline  (n = 22) | *Rrbp1*-KO  +  saline  (n = 27) | *Rrbp1*-KO  +  Fludrocortisone  (n = 18) |
| --- | --- | --- | --- |
| Blood | | | |
| [K^+^], mmol/L | 7.49 ± 0.10 | 8.19 ± 0.14‡ | 7.73 ± 0.16 |
| [Na^+^], mmol/L | 161.5 ± 1.10 | 163.6 ± 0.72 | 161.6 ± 0.88 |
| [Cl^-^], mmol/L | 124.6 ± 0.99 | 127.1 ± 0.77 | 124.6 ± 1.35 |
| BUN, mg/dL | 32.66 ± 1.09 | 35.32 ± 1.59 | 30.81 ± 1.91 |
| Cre, mg/dL | 0.28 ± 0.01 | 0.27 ± 0.01 | 0.27 ± 0.02 |
| Glu, mmol/L | 124.2 ± 6.10 | 117.3 ± 4.50 | 109.1 ± 5.10 |
| Urine | | | |
| Volume, ml | 0.86 ± 0.08 | 0.96 ± 0.06 | 1.79 ± 0.21‡ |
| K^+^, µmol | 1653 ± 157 | 1738 ± 100 | 3107 ± 342‡ |
| Na^+^, µmol | 897 ± 69 | 834 ± 46 | 1460 ± 143† |
| Cl^-^, µmol | 1210 ± 118 | 1322 ± 62 | 2368 ± 223‡ |
| BUN, mg | 21.7 ± 1.90 | 23.2 ± 1.20 | 33.7 ± 3.20† |
| BUN, Blood urea nitrogen; Cre, Creatinine; Glu, Glucose; †*P* < 0.01, ‡*P* < 0.001*Rrbp1*-KO (knock-out) vs. *Rrbp1*-WT (wild-type) | | | |


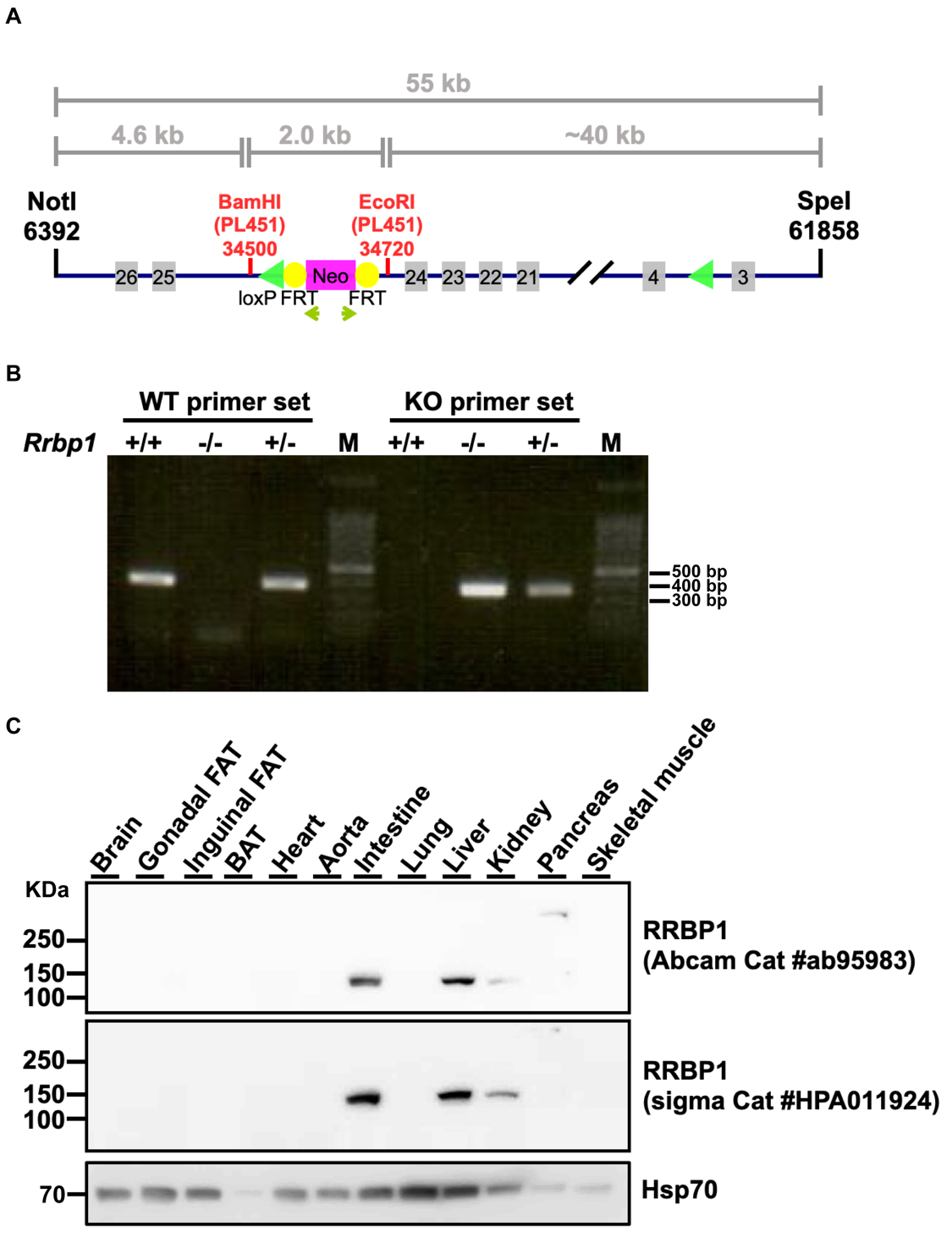


**Figure S1.** Generation of *Rrbp1*-KO mice. **A** Schematic representation of the knockout construct used for generating the *Rrbp1*-KO mouse line. Insertion of the FRT/ LoxP cassette in the intron between exons 3-4 and 24-25 disrupts *Rrbp1* mRNA expression. **B** Genotyping by PCR analysis of *Rrbp1*-WT (+/+), *Rrbp1*-HE (+/-), and *Rrbp1*-KO (-/-) mice. The *Rrbp1*-WT and *Rrbp1*-KO primer sets are expected to amplify the product of 400 bp. **C** Western blot analysis of the tissue distribution of RRBP1 recognized by two different commercial antibodies and Hsp70 protein in *Rrbp1*-WT mice. WT, wild-type; KO, knock-out; Neo, neomycin; Frt, flanked neomycin resistant gene.


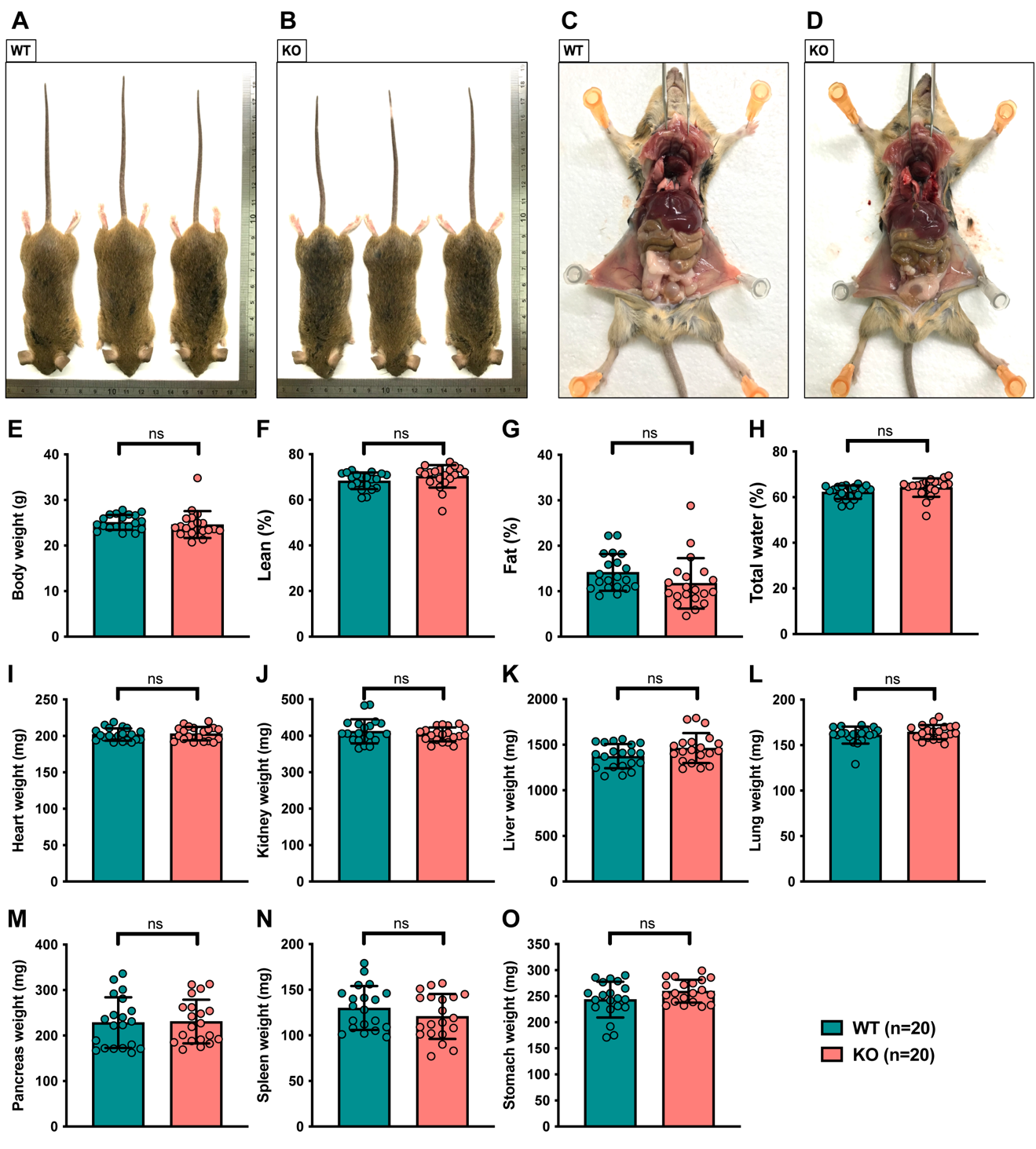


**Figure S2.** The basal phenotypes of Rrbp1-KO mice. **A, B** Morphology of *Rrbp1*-WT and *Rrbp1*-KO was observed at 16 weeks old. **C, D** Abdominal anatomy of *Rrbp1*-WTand *Rrbp1*-KO was dissected at 16 weeks old. **E-H** Total body weight, body lean, body fat, and body water of 16-week-old *Rrbp1*-WT and *Rrbp1*-KO mice. **I-O** Tissues weight of *Rrbp1*-WT and *Rrbp1*-KO mice. Heart; Kidney; Liver; Lung; Pancreas; Spleen; Stomach. ns, no significance;


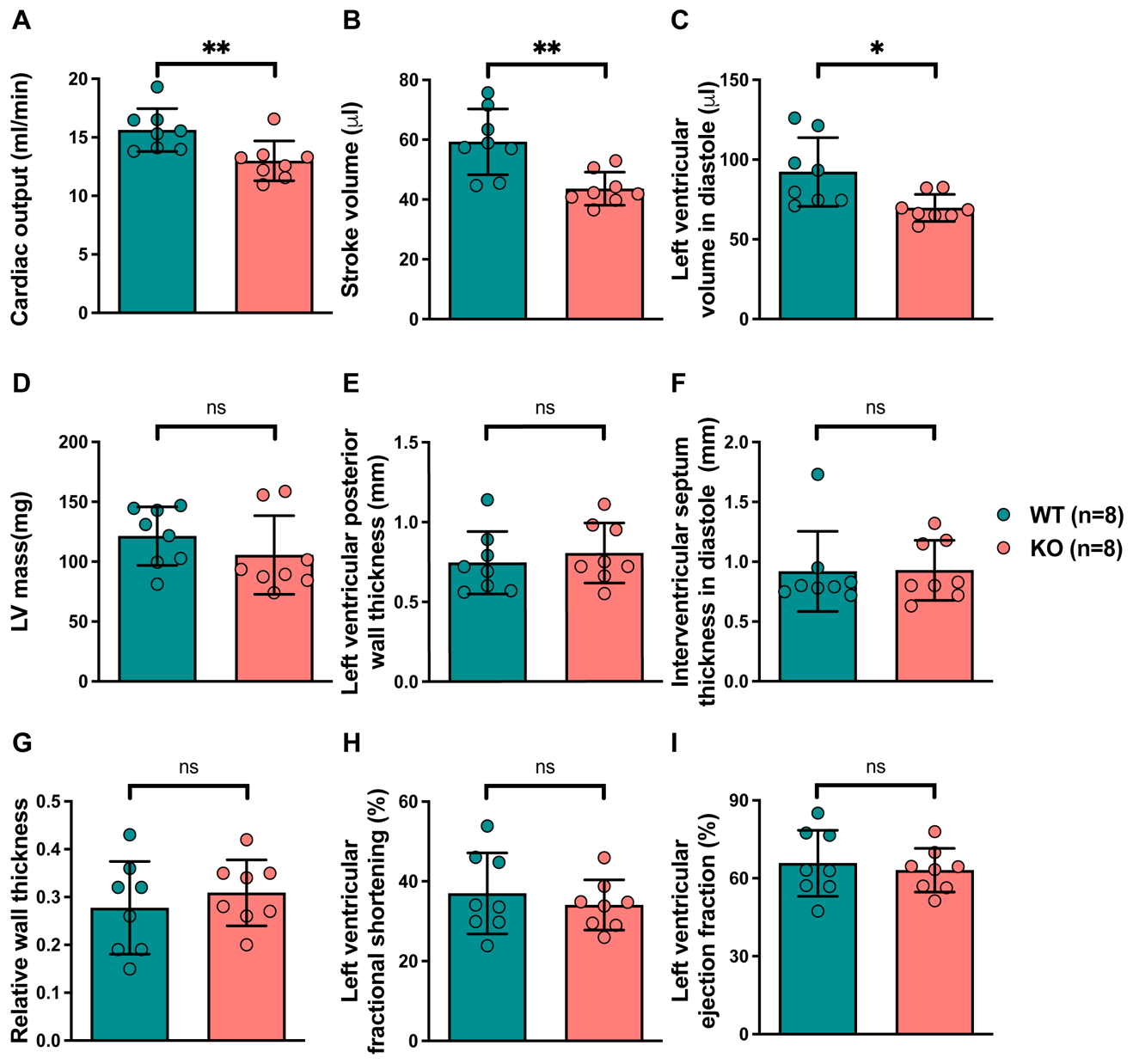


**Figure S3.** Echocardiographic measurement of mice with normal diet. **A-I** cardiac output; stroke volume; left ventricular volume in diastole; left ventricular mass; left ventricular posterior wall thickness; interventricular septum thickness in diastole; relative wall thickness in diastole; left ventricular fractional shortening; left ventricular ejection fraction measured by 2D-echocardiogram of *Rrbp1*-WT and *Rrbp1*-KO mice. (n=8 per group) WT, wild-type; KO, knock-out; LV, left ventricular. Data in (**A**)-(**I**) were analyzed with Student unpaired 2-tailed t test. Data were represented as mean ± SEM. ns, no significance; **P* < 0.05; ** *P* < 0.01


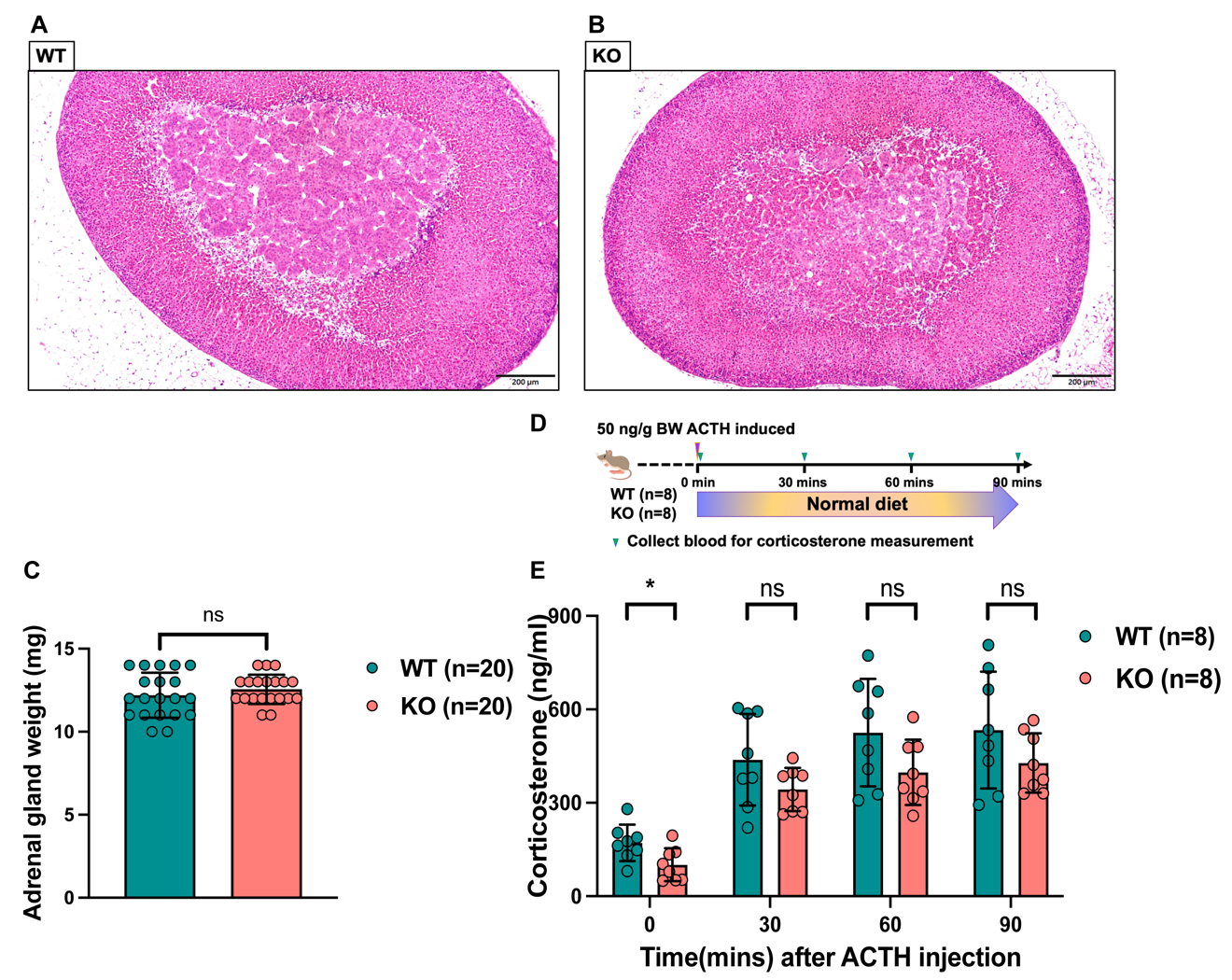


**Figure S4.** Dissection of adrenal gland function of *Rrbp1*-KO mice. **A, B** Hematoxylin and eosin staining of adrenal gland in *Rrbp1*-WTand *Rrbp1*-KO was observed at 16 weeks old. **C** Total adrenal gland weight of *Rrbp1*-WT and *Rrbp1*-KO mice. **D** Study protocol for ACTH stimulation test of mice. **E** Plasma corticosterone level of mice after ACTH treatment for 0, 30, 60, and 90 minutes. WT, wild-type; KO, knock-out Data in (**C**) and(**E**) were analyzed with Student unpaired 2-tailed t test. Data were represented as mean ± SEM. ns, no significance; **P* < 0.05


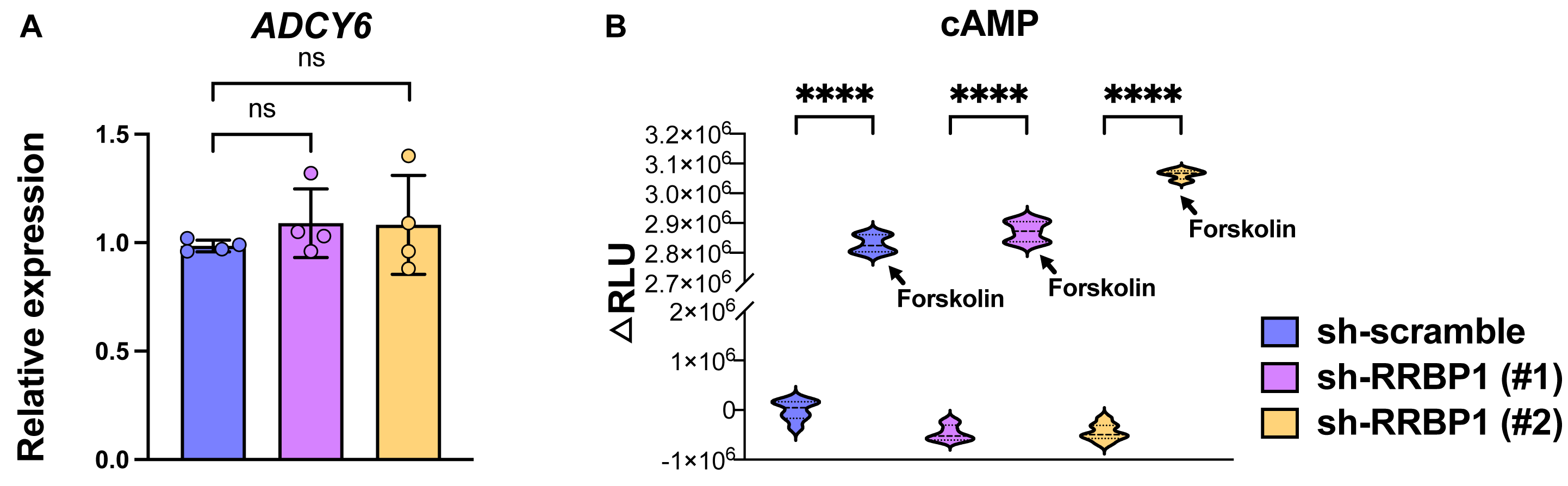


**Figure S5.** cAMP level with or without forskolin induction. **A** Intracellular mRNA levels of *ADCY6* in *RRBP1*-knockdown Calu-6 cells were measured using quantitative RT-PCR (qRT-PCR). Data were analyzed using the 2-ΔΔCt method with GAPDH as the reference gene. (n = 4 per group). **B** Intracellular cAMP levels in control and *RRBP1*-knockdown Calu-6 cells with or without 50 μM forskolin induction were determined by their relative luminescence. Dark arrow indicates the group with 50 μM forskolin induction. Each bar represents the mean ± SEM. n = 8 per group. Data in (**A**) were analyzed with Mann-Whitney test; data in (**B**) were analyzed with Student unpaired 2-tailed t test. Data were represented as mean ± SEM. ns, no significance; *****P* < 0.0001


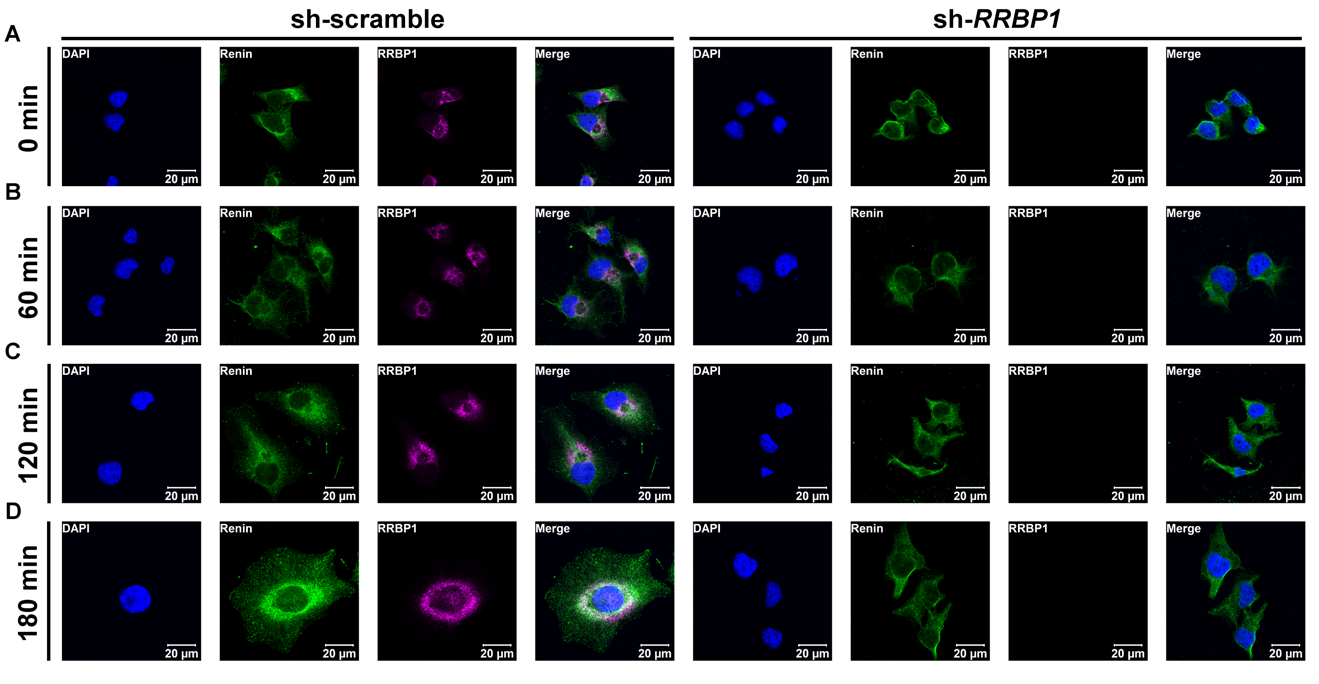


**FigureS6.**The distribution of renin and RRBP1 in control and *RRBP1*-knockdown cells with forskolin induction. **A–D** Representative confocal microscopy images of control and *RRBP1* knockdown Calu-6 cells showing renin (green), RRBP1 (magenta), and DAPI (blue) after 50 µM of forskolin induction for 0, 60, 120, and 180 minutes.


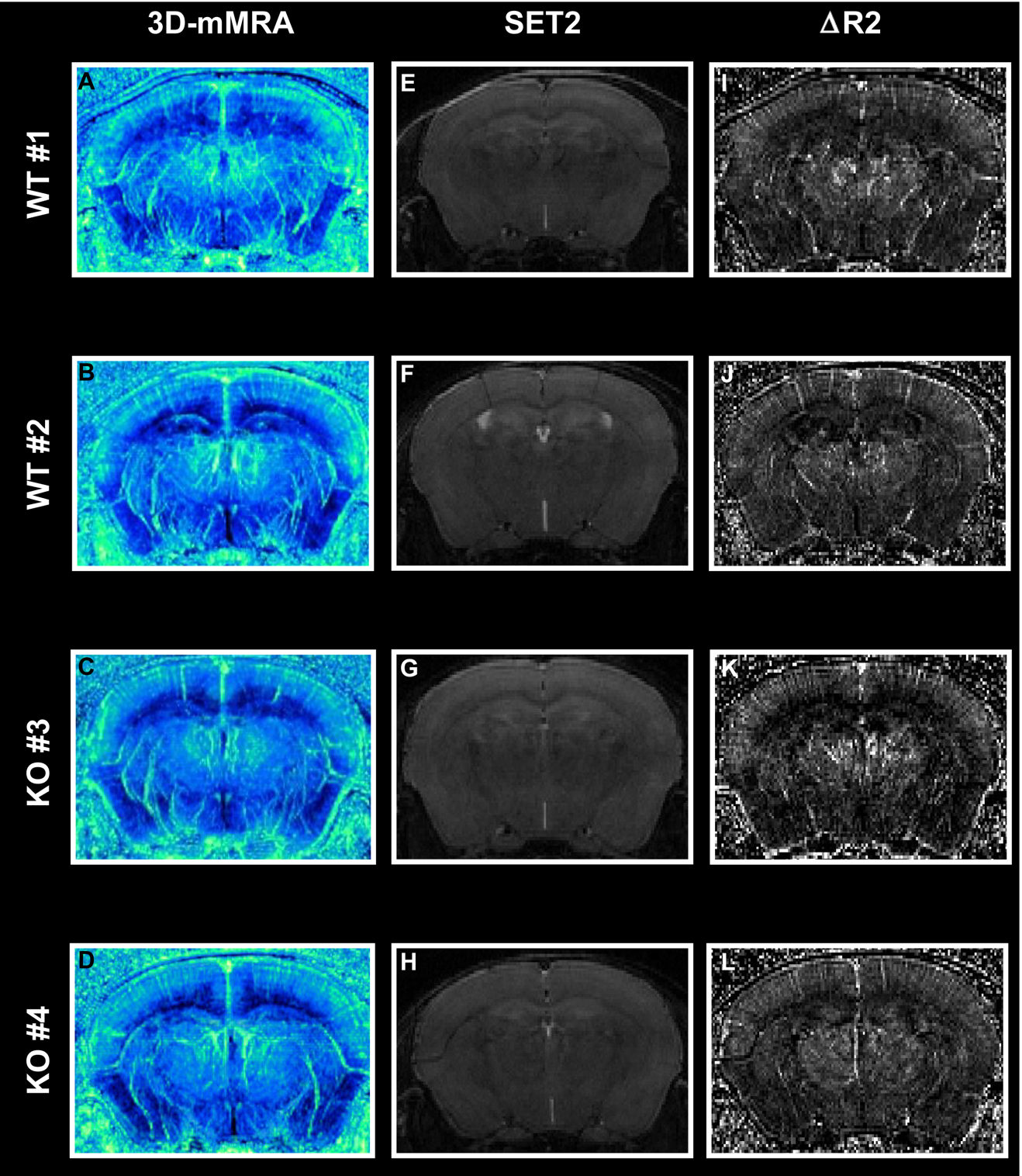


**Figure S7.** Intracranial vessel scanning of mice. **A-D** 3-dimentioanl-ΔR2 micro magnetic resonance angiography of *Rrbp1*-WT and *Rrbp1*-KO mice. **E-H** T1-weighted images (T1WIs) of *Rrbp1*-WT and *Rrbp1*-KO mice. **I-L** T2-weighted images (T2WIs) of *Rrbp1*-WT and *Rrbp1*-KO mice. WT, wild-type; KO, knockout.

**Supplemental Materials and Methods**

**The Stanford Asia-Pacific Program for Hypertension and Insulin Resistance (SAPPHIRe) cohort**

The Stanford Asia-Pacific Program for Hypertension and Insulin Resistance (SAPPHIRe) is a collaborative family study sponsored by the Family Blood Pressure Program of the National Heart, Lung and Blood Institute of the National Institutes of Health. The study was conducted to identify the genetic determinant of hypertension and insulin resistance in participants of Chinese ancestry. The study collected sibling pairs of over 1,144 participants from 360 nuclear families who were either concordant or discordant for high blood pressure as previously described [1]. The definition of high blood pressure was systolic blood pressure >140 mm Hg and diastolic blood pressure >90 mm Hg or with two medications for hypertension. Blood pressure in the bottom 30% of age- and sex-adjusted blood pressure distributions was defined as low-normal blood pressure. Individuals with heart, liver, or kidney diseases or chronic diseases such as diabetes or cancer were excluded. The study was approved by the Institutional Review Boards/Research Ethics Review Committee including National Taiwan University Hospital, National Health Research Institutes, Taichung Veterans General Hospital, Taipei Veterans General Hospital, and Tri-Service General Hospital. All participants signed informed consent. All procedures were conducted according to principles outlined in the Declaration of Helsinki.

**Genome-wide linkage and Family-based association analyses for identification of blood pressure regulation gene**

Genome-wide linkage analysis was conducted initially based on the 1,144 subjects of Chinese origin from 360 nuclear families of SAPPHIRe cohort. As a result, a quantitative trait locus (QTL) associated with variations of blood pressure located on chromosome 20 between 14.7-18.3 Mb was mapped. Detailed descriptions of linkage analysis were published in our previous work [1-3]. Further fine-mappings with 51 SNPs in 6 genes including *PCSK2*, *BFSP1*, *RRBP1*, *SNX5*, *OVOL2* and *CSRP2BP* were performed covering this QTL region. We applied family-based association tests (FBAT) for single-SNP and SNP-haplotype association analysis for these 51 SNPs. Sliding window approaches were performed to identify the haplotypes associated with blood pressure. Haplotype analyses and the permutation procedure were performed under the additive genetic model by the “hbat” module in FBAT.

**Animals**

For generation of *Rrbp1*-KO mice, two targeting vectors were generated to delete exons 4-24 of the *Rrbp1* gene by the recombineering-based method [4, 5]. The insertion of LoxP sites in the regions upstream of exon 4 and downstream of exon 24 within the *Rrbp1* gene were retrieved from the 129/Sv-derived bacterial artificial chromosome clones, bMQ361N11 and bMQ361N11 (Sanger Institute, UK), respectively. The two DNA fragments were inserted separately into a retrieving vector PL253, which contains an MC1-driven thymidine kinase cassette for negative selection of ES cells. The resulting constructs were used as the backbones for individual insertion of a neomycin (Neo) resistance cassette flanked by two LoxP sites upstream of exon 4, as well as a Neo cassette flanked by two Frt sites and one LoxP site downstream of exon 24, from PL452 and PL451 vectors, respectively. The first vector targeting exon 4 was linearized and transfected into 129/Sv-derived embryonic stem cells (R1) through electroporation. The correct targeted clones were confirmed using Southern blotting, and the neomycin resistance gene was removed using Cre expression. Subsequently, the resulting ES cells were transfected with the second vector targeting exon 24, followed by Southern blotting confirmation. After Cre-mediated gene deletion of *Rrbp1*, the selected ES cells were injected into C57BL/6 blastocysts to establish chimeric mice, and the founder male chimeras were then crossed into a C57BL/6 background (FigureS1*A*). *Rrbp1-*floxed mice were crossed with CMV-Cre mice to generate conventional knockout mice. Since the conventional knockout mice of C57BL/6 background were hard to obtain, C57BL6/129J mixed genetic background mice were generated by breeding B6 with 129J for up to 6 generations. Cross-breeding heterozygous with heterozygous of both C57BL6/129J mixed genetic background generated *Rrbp1*-WT (wild-type), *Rrbp1*-HE (heterozygotes), and *Rrbp1*-KO (homozygous knockout) mice for subsequent experiments. The genotyping results are shown in Supplemental FigureS1*B*.For genotyping, genomic DNA was extracted from 2-3 mm mouse tails and was used for PCR amplification by the wild-type primer set: forward primer 5’- AGAGAATGGTTGGGATAGAG -3’ and the reverse primer 5’- CAAACCCTTGTCATGAGCAT -3’ and the knockout primer set: forward primer 5’- CACTAC ATGTGTAGCTGAAC -3’ and the reverse primer 5’- CAAACCCTTGTCATGAGCAT-3’.

For high-potassium loading mouse survival test, mice were under high K^+^ intake and intraperitoneally injected with fludrocortisone acetate once every two days for a total of 30 days. The death events were recorded.

Mice were sacrificed for tissue dissection or euthanized by excess carbon dioxide administration. All the animal protocols and experimental procedures were approved by the Institutional Animal Care and Utilization Committee, Academia Sinica (IACUC number: 19-07-1331) and performed in conformation with the National Institutes of Health (NIH) guidelines for the Care and Use of Laboratory Animals.

**Phenotypic analysis**

Body composition analysis: The body compositions of 16-week-old mice were determined by the SkyScan1076 high resolution micro-CT system. Mice were anesthetized using 1% isoflurane inhalation. We thank the Taiwan Mouse Clinic, Academia Sinica and Taiwan Animal Consortium for the technical support in standard body composition analysis.

Echocardiogram: Echocardiography was performed on mice at age 12-16 weeks with normal diet using a preclinical ultrasound system with a linear 30-MHz transducer (Prospect T1, S-Sharp Corporation). Mice were anesthetized with 2% isoflurane (200 ml/min air) during imaging. Cardiac output (ml/min) was calculated using the heart rate (number of heart beats per minute) multiplied by stroke volume (ml/beat): Cardiac output = heart rate × stroke volume. Left ventricular mass was calculated from M-mode images using interventricular septum, diastolic left ventricular diameters of left ventricular internal diameter and left ventricular posterior wall for the formula corrected by Devereux: LV mass (mg) = 1.04 × [(interventricular septum + left ventricular internal diameter + left ventricular posterior wall thickness)^3^– (left ventricular internal diameter)^3^] – 13.6.

Blood and urine analysis: Ten to sixteen-week-old male and female mice were used. For high potassium loading test or the fludrocortisone acetate treatment assay, mice were placed in metabolic cages at 11:00 am. They were first fed with control diet (D10012Mi, Research Diets) and normal water for 24 hours, followed by high-K^+^ diet (5% potassium was added as potassium chloride) and water containing 5% KCl for another 48 hours. For fludrocortisone acetate treatment assay, mice were intraperitoneally injected with saline or 100 mg/kg fludrocortisone acetate (F6127, Sigma) after high-K^+^ intake for 24 hours. After another 24 hours, urine and blood from the submandibular vein were collected and examined. The electrolytes profile was analyzed by different kits of cobas c11 analyzer (Roche). The plasma samples with hemolysis index from zero to “++” (indicated very mild level of hemolysis) were obtained to conduct electrolytes measurement.

Commercial ELISA kits were utilized to measure the levels of renin (E-EL-M0061, Elabscience), angiotensinogen (CSB-E08566m, Cusabio), Ang-I (CSB-E08529m, Cusabio), Ang-II (CSB-E04495m, Cusabio), aldosterone (ADI-900-173, Enzo Life), and noradrenaline (CSB-E07870m, Cusabio). The renin activity assay was performed using SensoLyte®520 Mouse Renin Assay Kit (AS-72161, AnaSpec). Ten µl serum sample was utilized to measure the mouse renin activity using a 5-FAM/QXL™ 520 fluorescence resonance energy transfer peptide. The FRET peptide was separated into two fragments by mouse renin, and the fragments of 5-FAM were monitored at excitation/emission = 490/520 nm.

The fractional excretion of electrolytes was calculated using the following equation: FE = (serum creatinine (μmol/L) × urinary electrolyte (mmol/L)/serum electrolyte (mmol/L) × urinary creatinine (μmol/L) × 100.The transtubular K^+^ gradient (TTKG) was calculated using the formula: TTKG= (urinary potassium × serum osmolality)/ (serum potassium × urine osmolality). The plasma osmolality was calculated using the formula: Osmolality= 2 × serum sodium(mEq/L) + glucose (mg/dL)/18+BUN (mg/dL)/2.8.

ACTH stimulation test: 16-week-old mice were intraperitoneally injected with 50 ng/g body weight of Adrenocorticotropic Hormone (ACTH 1-24; A0928, Sigma) at 11:00. Then, we collected blood sample after ACTH treatment for 0, 30, 60, and 90 minutes. Commercial ELISA kits were utilized to measure the levels of corticosterone (CSB-E07969m, Cusabio).

Immunohistochemistry: Mouse kidneys and adrenal gland were paraffin-embedded and 4-µm tissue sections were cut from the paraffin blocks on the slides. Adrenal gland sections were undergone standard Hematoxylin and eosin staining. Kidney sections were rehydrated by immersing in following xylene, 100%, 95%, 70%, and 50% alcohol. Sections were immersed into 1 mM Citrate acid buffer pH 6.0 with tween 20 and heated to 92-95 °C for antigen retrieval. To quench endogenous peroxidase activity, sections were incubated within 3% H_2_O_2_ for 20 minutes. Tissue was blocking within 3% BSA/PBST. Immunochemistry staining was conducted using primary antibody for renin (1:20; H0005972-M01, Abnova), secondary antibodies HRP-anti-rabbit/mouse (K5007, Dako), and DAB chromogen solution. The images digitized using an advanced microscope (Axio Imager. A1, Zeiss).

Intracranial vessel scanning by magnetic resonance angiography: All imaging was performed using a 7-T scanner (PharmaScan 70/16, Bruker Biospin GmbH, Germany) with an actively shielded gradient capable of producing a maximum gradient amplitude of 300 mT/m with an 80 µs. The mice were initially anesthetized with 5 % isoflurane in air at a flow rate of 1 L/min. When fully anesthetized, the mice were placed in a prone position and fitted with a custom-designed head holder inside the magnet. Anesthesia was then maintained with 1.0–1.2 % isoflurane in air at a flow rate of 1 L/min throughout the experiments. Images were acquired using a 38-mm birdcage transmitter coil and a separate quadrature surface coil for signal detection. To determine 3-dimentioanl- ΔR2 micro magnetic resonance angiography (3DΔR2−μMRA) [6, 7], T_2_-weighted images (T2WIs) were acquired before and after the animals were injected with superparamagnetic iron oxide nanoparticles at a dose of 20 mg/kg. The postcontrast image acquisition was delayed by 1-2 min to ensure that the distribution of the contrast agent in the vascular network had reached a steady state. T2WIs were acquired using a 3D fast spin echo (FSE) sequence with a repetition time of 1,800 ms, an effective echo time (TE) of 80 ms, 2 averages, a field of view of 2.0 × 2.0 × 1.0 cm, an acquisition matrix of 512 × 192 × 96, and a total acquisition time of 1 h 9 min.

***In vitro* studies**

Calu-6 cell culture: The human renin-secreting Calu-6 cell line was purchased from the ATCC (HTB-56^TM^). Calu-6 cells were cultured in Dulbecco’s modified Eagle medium/nutrient mixture F12 supplemented with 10% fetal bovine serum (FBS) and 1% penicillin–streptomycin (p4333, Sigma), and were maintained at 37°C with a 5% CO2-containing humidified incubator. Cells were cultured in 10-cm plastic tissue culture dishes (430167, Corning Inc.) routinely and harvested with trypsin upon reaching a log phase of growth.

Lentiviral transfection: Lentiviral knockdown *RRBP1* and lentiviral scramble control particles were purchased from RNAi (Academia Sinica, Taiwan) core. For cell infection, 45-55% confluent Calu-6 cells were incubated with lentiviral particles and 24 μg polybrene in 3-ml growth medium at a multiplicity of infection of 10. After 24 hours, the medium was replaced with 4-ml fresh growth medium. After 48 hours, the medium was replaced with 4-ml fresh growth medium and 8 μg puromycin (A1113802, Thermo Fisher) for cell selection. After five days, all transfected Calu-6 cells were passaged for other experiments. The *RRBP1*-knockdown efficiency was examined using qPCR and western blotting analyses.

Western blot assay: Mouse tissues and cell lysates were extracted using the RIPA lysis buffer (20-188, Millipore) according to the manufacturer’s protocol. The culture medium of cells was collected and concentrated by Amicon Ultra-15 tubes (10 KDa, Millipore). Protein concentrations in samples were determined using the Bradford protein assay (500006, BioRad). Protein samples were separated using 10% sodium dodecyl sulfate polyacrylamide gel electrophoresis and transferred onto a nitrocellulose membrane. The membranes were first blocked by incubating in Tris-buffered saline with 0.05% Tween-20 and 5% fat-free milk, and then incubated with different primary antibodies in Tris-buffered saline with 0.05% Tween-20 and 5% fat-free milk overnight at 4°C. The following antibodies were used: anti-RRBP1 (PA5-21392, Invitrogen), anti-RRBP1 (Ab95983, Abcam), anti-RRBP1 (HPA011924, Sigma), anti-Hsp70 (DF2698, Affinity), anti-β-actin (GTX109697, Genetex), anti-renin (H0005972-M01, Abnova), anti-ACE (MA5-32741, Invitrogen), anti-β-tubulin (tcaba2, Taiclone), anti-SGK1 (ab32374, Abcam), anti-ADCY5/6(PA5-75274, Abnova), anti-calnexin (ab22595, Abcam). After washing the membranes three times with 0.08% Tween 20-Tris-buffered saline, they were incubated with a horseradish peroxidase-conjugated secondary antibody (1:5000, Thermo) at room temperature for 2 hours. The blots were detected by Trident pico Western HRP Substrate solution (GTX17435, Genetex) and the images were analyzed using a UVP BioSpectrum Auto Imaging System.

Real-time quantitative PCR (RT-qPCR): RNA samples were extracted using TRIzol® reagent (Life Technologies). Total RNA (2µg) was reverse transcribed to cDNA using reverse transcriptase (HD life science). The RT-qPCR assay was conducted using SYBR-Green qPCR mix (HD life science) and performed by LightCycler® 480 System Real-Time PCR System (Roche). The primer sequences are as follows: *RRBP1*, forward 5′-TACGACACTCAAACCTTGGGG-3′ and reverse 5′-GGTTGGCTAGGGCTTCTTCATA-3′; *ADCY6*, forward 5′-GCTCATGGTGGTGTGTAACC-3′ and reverse 5′-GCGTGTAGGCGATGTAGACAAA-3′;*Scnn1a*, forward 5′-CCTTCTCCTTGGATAGCCTGG-3′ and reverse 5′-CAGACGGCCATCTTGAGTAGC-3′; Scnn1b, forward 5′-GGCCCAGGCTACACCTACA-3′ and reverse 5′-AGCAGCGTAAGCAGGAACC-3′; Scnn1g, forward 5′-GCACCGACCATTAAGGACCTG-3′ and reverse 5′-GCGTGAACGCAATCCACAAC-3′; *GAPDH*, forward 5′-CTGGGCTACACTGAGCACC-3′ and reverse 5′-AAGTGGTCGTTGAGGGCAATG-3′.

Intracellular cAMP measurement: Calu-6 cells were transferred into serum-free medium containing 50 μM forskolin (100-0249, STEMCELL Inc.) or DMSO as control when cells reached 70% confluence. After incubation with forskolin or DMSO for 16 hours, cAMP levels were measured using cAMP-Glo^TM^ assay (V1501, Promega) and the luminescence was read with a plate-reading luminometer.

Immuno-electron microscopy: Cells were plated on plastic coverslips in 60-mm tissue culture plates. For two days, cells were fixed with 2% paraformaldehyde and 1% glutaraldehyde in 0.1 M phosphate buffer (pH7.4) for 40 minutes. Samples were blocked and permeabilized with PBS containing 5% BSA and 0.1% saponin for 30 minutes, followed by incubation with primary antibodies for renin (1:20; H0005972-M01, Abnova) in PBS containing 5% BSA and 0.05% saponin for 2 hours at room temperature. After washing with PBS 4 times, samples were incubated with secondary antibody (1:100; 2002 Nanogold^®^-Fab, Nanoprobed) in PBS containing 5% BSA and 0.05% saponin for 1 hour at room temperature, followed by fixation with 2%glutaraldehydein PBS for 30 minutes at room temperature. Silver enhancement of samples were conducted with the HQ kit from Nanoprobes. The samples were then washed with deionized water 2 times and postfixed with 1% osmium tetroxide and dehydrated with increasing percent solutions of ethanol and embedded in 100% epoxy resin. Ultrathin sections were cut at 70 nm thicknesses, and stained with 1% uranyl acetate for 15 minutes and 3% lead citrate for 5 minutes. Sections were examined using a Philips CM 100 Transmission Electron Microscope at 80 KV and the GatanOrius CCD camera was utilized for obtaining the images.

Immunofluorescence stain: The cultured cells on cover slips were fixed in 10% formaldehyde solution (HT501128, Sigma) for 10 minutes at room temperature, followed by washing thrice with PBS for 5 minutes each. Then, cells were permeabilized with 0.1% TritonX-100 (108603, Merck) for 10 minutes at room temperature, followed by washing thrice with PBS for 5 minutes each. The cells were blocked for 30 minutes in PBS containing 1% BSA and then incubated overnight at 4°C with the following primary antibodies in the blocking buffer: mouse anti-renin (1:50, H00005972-M01, Abnova); rabbit anti-calnexin (ab22595, Abcam); rabbit anti-GOLIM4 (PAB28477, Abnova); rabbit anti-TGN46 (NBP1-49643, NovusBio) followed by Alexa Fluor^®^ 488 goat anti-mouse IgG_(H+L)_ (1:100, A11029, Thermo Fisher) or Alexa Fluor^TM^ 555 goat anti-rabbit IgG_(H+L)_ (1:100, A21428, Thermo Fisher) secondary antibody for 1.5 hours at room temperature. Then, slides were mounted with a drop of DAPI Fluoromount-G^®^ (0100-20, Southern Biotech). Samples were examined using a laser scanning confocal microscope Zeiss LSM 700. Confocal images were acquired with a 63× oil objective lens in a 1024 × 1024 pixels format at a 12-bit intensity resolution.

**Statistics**

All data represent as mean ± standard error of the mean (SEM). Data set which compared three independent groups (blood pressure of Rrbp1-WT, HE, and KO mice) is subjected to ordinary one-way ANOVA test. The Kaplan-Meier survival curve and log-rank test was applied for survival analysis. For datasets which were followed normal distribution, comparisons were made by unpaired, two-tailed Student’s t-test (p ≤ 0.05). If datasets were not followed normal distribution, comparisons were created using nonparametric tests, two-tailed Mann-Whitney U test (p ≤ 0.05). All representative images were used from one of the repeat experiments that best matched the average data in each assay.

**Supplemental references**

1. Liu P, Jenkins NA, Copeland NG. A highly efficient recombineering-based method for generating conditional knockout mutations. Genome Res. 2003;13(3):476-484.
2. Chuang LM, Chiu YF, Sheu WH, et al. Biethnic comparisons of autosomal genomic scan for loci linked to plasma adiponectin in populations of Chinese and Japanese origin. J Clin Endocrinol Metab. 2004;89(11):5772-5778.
3. Lange C, Silverman EK, Xu X, Weiss ST, Laird NM. A multivariate family-based association test using generalized estimating equations: FBAT-GEE. Biostatistics. 2003;4(2):195-206.
4. Shinagawa T, Do YS, Baxter J, Hsueh WA. Purification and characterization of human truncated prorenin. Biochemistry. 1992;31(10):2758-2764.
5. Liu P, Jenkins NA, Copeland NG. A highly efficient recombineering-based method for generating conditional knockout mutations. Genome Res. 2003;13(3):476-484.
6. Huang CH, Shih YY, Siow TY, et al. Temporal assessment of vascular reactivity and functionality using MRI during postischemic proangiogenenic vascular remodeling. MagnReson Imaging. 2015;33(7):903-910.
7. Hsiao HY, Chen YC, Huang CH, et al. Aberrant astrocytes impair vascular reactivity in Huntington disease. Ann Neurol. 2015;78(2):178-192.
